# IL6-STAT3-C/EBPβ-IL6 positive feedback loop in tumor-associated macrophages promotes the EMT and metastasis of lung adenocarcinoma

**DOI:** 10.1186/s13046-024-02989-x

**Published:** 2024-02-29

**Authors:** Zhengyang Hu, Qihai Sui, Xing Jin, Guangyao Shan, Yiwei Huang, Yanjun Yi, Dejun Zeng, Mengnan Zhao, Cheng Zhan, Qun Wang, Zongwu Lin, Tao Lu, Zhencong Chen

**Affiliations:** 1 Department of Thoracic Surgery, Zhongshan Hospital, Fudan University, No. 180, Fenglin Road, Shanghai, 200032 China; 2 Department of Thoracic Surgery, Shanxi Province Cancer Hospital / Shanxi Hospital Affiliated to Cancer Hospital, Chinese Academy of Medical Sciences / Cancer Hospital Affiliated to Shanxi Medical University, No. 3 Gongren Xin Jie, Xinghualing District, Taiyuan, 030013 Shanxi Province China

**Keywords:** Lung adenocarcinoma, Tumor-associated macrophages, Metastasis, Interleukin-6, Epithelial-mesenchymal transition

## Abstract

**Background:**

Lung cancer is one of the most common tumors in the world, and metastasis is one of the major causes of tumor-related death in lung cancer patients. Tumor-associated macrophages (TAMs) are a major component of the tumor microenvironment (TME) and are frequently associated with tumor metastasis in human cancers. However, the regulatory mechanisms of TAMs in lung cancer metastasis remain unclear.

**Methods:**

Single-cell sequencing analysis of lung cancer and normal tissues from public databases and from 14 patients who underwent surgery at Zhongshan Hospital was performed. In vitro co-culture experiments were performed to evaluate the effects of TAMs on lung cancer migration and invasion. Changes in the expression of IL-6, STAT3, C/EBPΒ, and EMT pathway were verified using RT-qPCR, western blotting, and immunofluorescence. Dual luciferase reporter assays and ChIP were used to reveal potential regulatory sites on the transcription factor sets. In addition, the effects of TAMs on lung cancer progression and metastasis were confirmed by in vivo models.

**Results:**

TAM infiltration is associated with tumor progression and poor prognosis. IL-6 secreted by TAMs can activate the JAK2/STAT3 pathway through autocrine secretion, and STAT3 acts as a transcription factor to activate the expression of C/EBPβ, which further promotes the transcription and expression of IL-6, forming positive feedback loops for IL6-STAT3-C/EBPβ-IL6 in TAMs. IL-6 secreted by TAMs promotes lung cancer progression and metastasis in vivo and in vitro by activating the EMT pathway, which can be attenuated by the use of JAK2/STAT3 pathway inhibitors or IL-6 monoclonal antibodies.

**Conclusions:**

Our data suggest that TAMs promote IL-6 expression by forming an IL6-STAT3-C/EBPβ-IL6 positive feedback loop. Released IL-6 can induce the EMT pathway in lung cancer to enhance migration, invasion, and metastasis. The use of IL-6-neutralizing antibody can partially counteract the promotion of LUAD by TAMs. A novel mechanism of macrophage-promoted tumor progression was revealed, and the IL6-STAT3-C/EBPβ-IL6 signaling cascade may be a potential therapeutic target against lung cancer.

**Graphical Abstract:**

IL-6 secreted by TAM acts on itself to promote STAT3 phosphorylation, and pSTAT3 transfers into the nucleus, promotes the expression of C/EBPβ. C/EBPβ is able to further promote IL-6 expression, which forms positive feedback for IL-6 secretion. IL-6 secreted by TAMs acts on lung cancer to promote their metastasis through activation of EMT.
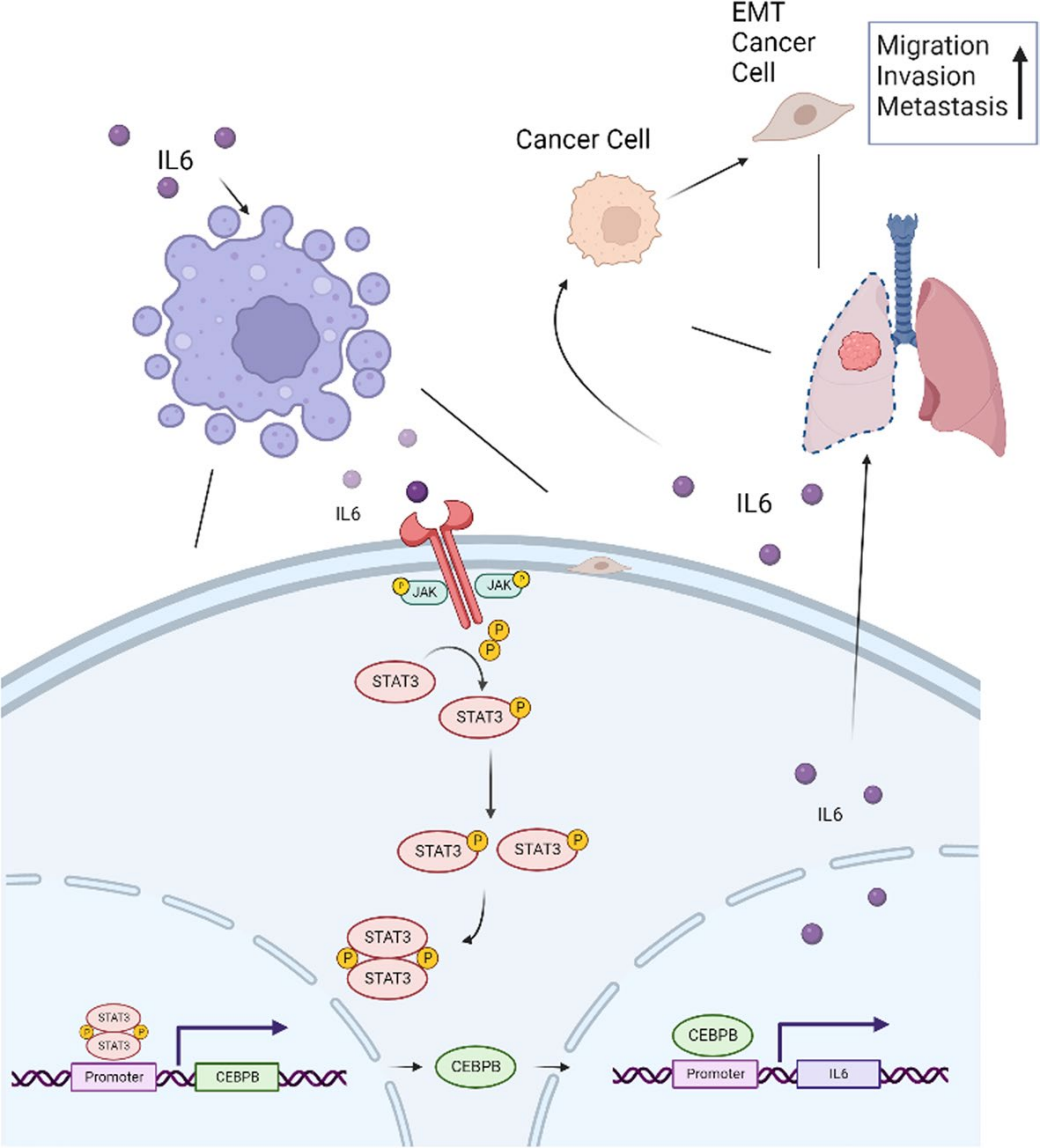

**Supplementary Information:**

The online version contains supplementary material available at 10.1186/s13046-024-02989-x.

## Background

Lung cancer is the second most common malignancy and the leading cause of cancer-related mortality worldwide [1]. With the introduction of novel treatments including immunotherapy and targeted therapy, the 5-year survival rate for advanced lung cancer has increased from less than 5% to over 30% [2, 3]. Despite this, metastasis continues to be a factor in tumor-related mortality in lung cancer patients and brings critical challenges for treatment [4]. Additionally, despite curative resection, between 30 and 55% of postoperative NSCLC patients may have metastases and recurrence, and show poor survival [5, 6]. Therefore, elucidating the mechanisms of metastasis is crucial for the development of effective lung cancer therapies.

The tumor microenvironment (TME) is the basis for tumor survival and plays an important role in tumorigenesis, progression, and treatment [7, 8]. The tumor microenvironment is also a very heterogeneous tissue that contains cells, including tumor cells, stromal cells, immune cells, and other cell types [9]. Macrophages in the TME, also referred to as tumor-associated macrophages (TAMs), are usually among the most common infiltrating cell types in the solid tumor microenvironment. Traditionally, based on the state of activation, TAMs in vivo have been classified as ‘classically activated’ M1 or ‘alternatively activated’ M2 macrophages [10]. However, TAMs exhibit a wide variety of phenotypes and functions, and are frequently thought to be linked to poor prognosis in patients with various tumors, such as breast, renal, gastric, and lung cancer [11–14]. In the TME, on the one hand, tumor cells recruit macrophages into the tumor mesenchyme and promote TAMs polarization toward the M2 type by releasing cytokines and exosomes [15, 16]. On the other hand, TAMs can also activate the epithelial mesenchymal transition (EMT) in tumors through chemokines and cytokines, such as TGF-β, IL-10, IL-6, matrix metalloproteinases (MMPs), and vascular endothelial growth factor (VEGF), to promote tumor invasion and migration [17–20].

Interleukin-6 (IL-6) is an important proinflammatory cytokine that is released during infection or tissue injury and promotes innate and acquired immune responses [21]. In the TME, IL-6 is closely associated with tumorigenesis, EMT, and tumor metastasis [22, 23]. In addition, IL-6 has been associated with lung cancer resistance to chemotherapy, and high serum IL-6 expression in lung cancer patients after chemotherapy is associated with a worse prognosis [24, 25]. Drugs targeting IL-6 are gradually being used in the clinic to treat cancer [26].

Here, we identified a microenvironment with high IL-6 expression in lung cancer, and TAMs are one of the major sources of IL-6. By stimulating the JAK2/STAT3-C/EBPβ-IL6 pathway, IL-6 can promote the release of additional IL-6, creating a positive feedback loop in TAMs; thus, it promotes EMT, invasion, and migration of lung cancer in vivo and in vitro. In contrast, TAM-induced lung cancer metastases were greatly reduced when neutralizing antibodies against IL-6 were uesd.

## Materials and methods

### Single-cell RNA sequencing data collection

The study conducted scRNA-seq on 14 patients who were treatment-naive and were diagnosed with lung adenocarcinoma (LUAD) through postoperative pathology at Zhongshan Hospital, Fudan University (including two advanced LUAD and 12 early LUAD samples). In addition, seven normal and three advanced LUAD samples and five normal samples were obtained from ArraryExpress (accession numbers EMTAB- 6149 and E-MTAB-6653) and Human Cell Atlas Data Coordination Platform (accession number PRJEB31843), respectively. The clinical characteristic of patients was shown in the Supplementary Table 4.

### ScRNA-seq data processing

Dimensionality reduction, clustering, and subclustering of single-cell datasets are implemented through the Seurat package, which was described in our previous study [14]. Differential gene expression analysis between the two groups was achieved by the FindAllMarkers function in Seurat.

### Immunofluorescence assay

Immunofluorescence of tissue sections was first blocked using 3% H_2_O_2_, followed by antigen repair using EDTA buffer (pH = 9.0). After cooling to room temperature, 0.2% tritonX-100 was added to break the membrane for 15 min. Blocked with FBS for 15 min, followed by the addition of CD68 (Abcam, ab201340, 1:100), CD163 (Abcam, ab182422, 1:500), IL-6 (Absin, abs135607, 1:200) antibodies and incubated overnight at 4 °C. After PBS wash, added fluorescent secondary antibody and incubated for 1 hour at room temperature CD68 (ThermoFisher, A21202, 1:400), CD163 (ThermoFisher, A21207, 1:600), IL-6 (ThermoFisher, A31573, 1:400). Add DAPI and incubate for 15 min at room temperature and protect from light. Seal the slices with an anti-fluorescence quencher.

Immunofluorescence of the cells was first performed by fixing the slide with 4% paraformaldehyde for 15 min. The slide was permeabilized with 0.5% Triton X-100 at room temperature for 20 min. Goat serum was added and blocked at room temperature for 30 min. pSTAT3 (Cell Signaling Technology, 4113S, 1:100) and C/EBPβ (Cell Signaling Technology, 43095S, 1:400) antibody was added and incubated overnight at 4 °C. Wash the slide with PBST and add the fluorescent secondary antibody, anti-mouse (Beyotime, A0428, 1:100), anti-rabbit (Beyotime, A0468, 1:100) and incubate for 1 h at room temperature. The slides are sealed with a blocking solution containing an anti-fluorescence quencher.

### Survival analysis

The data used for survival analysis were obtained from The Cancer Genome Atlas (TCGA) database (https://www.cancer.gov/ccg/research/genome-sequencing/tcga). The R package “survival” was used to generate Kaplan-Meier plots for overall survival (OS) and disease-free survival (DFS). *P* < 0.05 was considered a difference in survival time.

### Transwell assay

For cell migration assays, 24-well Transwells (8 μm pore size, Corning, NY, USA) were used. For cell invasion assays, 24-well Transwells with Matrigel (BD Biosciences, CA, USA) were used. 10^4^ macrophage cells were resuspended in 500 μl medium and added to the lower chamber. Then, 10^4^ A549 or H358 cells were resuspended in 200 μl serum-free medium and added to the upper chamber. After 24-48 hours, the chambers were removed and fixed using 4% paraformaldehyde for 10 minutes, followed by crystal violet solution staining for 15 minutes. Excess crystal violet solution was washed away using PBS and air dried. Five fields of view were then selected for photography and cell counting was performed using ImageJ.

### CCK8 assay

Add 100 μl of cell suspension containing 2000 A549 or H358 cells to a 96-well plate. Add 10 μl CCK8 solution (Dojindo, Kumamoto, Japan), incubate for 2 hours at 37 °C, and measure the absorbance at 450 nm with an enzyme marker. Cell viability = [(Experimental hole - Blank hole) / (Control hole - Blank hole)] × 100%.

### RNA extraction and RT-qPCR

RNA was extracted with TRIzol, followed by reverse transcription using the PrimeScript RT Reagent kit (TaKaRa, Tokyo, Japan). RT-qPCR analysis was performed using SYBR Green Premix Ex Taq (TaKaRa, Tokyo, Japan) and the QuantStudio 5 Real-Time PCR System (Thermo Fisher Scientific, MA, USA) was utilized to measure relative RNA expression levels. Relative expression was calculated using the 2-ΔΔCt method. The primer sequences were provided in Supplementary materials.

### Enzyme-linked immunosorbent assay (ELISA)

IL-6 was used to stimulate macrophages, and the medium was changed after 4 hours. At specified time points, 20 μl of the cell culture supernatant was collected and diluted 1:4 with PBS. The IL-6 Elisa test kit was purchased from Dakewe (Cat No:1110602, Beijing, China). Add 100 μl of sample and standard to the wells, and set up 3 replicate wells for each sample. Add 50 μl of Biotinylated antibody and incubate for 1 hour at room temperature. Discard the fluid in the plate, add 300 μl washing buffer to each well, and repeat 3 times. Add 100 μl Streptavidin-HRP to each well and incubate at room temperature for 20 minutes. Add 100 μl TMB, and incubate for 15 minutes at room temperature, protected from light. Terminate the reaction by adding 100 μl Stop solution to each well. Read the absorbance at 450 nm with an enzyme marker.

### Cell culture and macrophage polarization

The lung adenocarcinoma cell lines (A549, H358), lung epithelial cell line (BEAS-2B), and monocytic cell lines (THP1, RAW264.7) were purchased from the Chinese Academy of Sciences Cell Bank. The lung adenocarcinoma cells and lung epithelial cells were cultured in DMEM (Hyclone, UT, USA), supplemented with 10% fetal bovine serum (Hyclone, UT, USA), 1% penicillin, and 1% streptomycin (Beyotime, Shanghai, China). Monocytic and macrophage cell lines were cultured in RPMI 1640 (Hyclone, UT, USA), supplemented with 10% fetal bovine serum (Biosun, Australia), 1% penicillin, and 1% streptomycin (Beyotime, Shanghai, China). The culture was maintained in a 5% CO2 atmosphere at 37 °C with saturated humidity. THP1 polarized towards to M2-like macrophages by first adding 100 ng/ml PMA (Sangon Biotech, A606759, Shanghai, China) to stimulate for 24 hours. Subsequently, 25 ng/ml IL4 (Sino Biological, 11846-HNAE, Beijing, China) and 25 ng/ml IL13 (Sino Biological, 10369-HNAE, Beijing, China) were added to stimulate for 48 hours. RAW264.7 cells were polarized toward M2-like macrophages by adding 20 ng/ml mouse-derived IL4 (Sino Biological, 51,084-MNAE, Beijing, China) and stimulated for 24 hours. BMDM cells were obtained from 6-week-old C57BL/6 mice. Tibia and femur were isolated after execution and sterilization of mice, and bone marrow cells were flushed using culture medium. Erythrocytes were removed using erythrocyte lysate (Beyotime, Shanghai, China), followed by centrifugation at 1000 rpm for 10 min, discarded the supernatant. Cells were resuspended using DMEM medium. After 1 day, cells were adhered to the wall and DMEM complete medium containing 10 ng/ml of M-CSF (Sino Biological, 10369-HNAE, Beijing, China) was added to induce myeloid cells to differentiate into macrophages. After 5 days, 20 ng/ml of mouse-derived IL-4 was added to polarise towards M2-like macrophages.

### Flow cytometry

After polarization of THP1 cells into M2-like macrophages, cells were digested using Accutase (BioLegend, 423201). After centrifugation, cells were resuspended using 100 μl PBS, and incubated for 5-10 min at 4 °C using FcRblock (1 μg/test, 101319, BioLegend). Subsequently, staining was performed using APC anti-human CD11b (0.5 μg/test, 101212, BioLegend), PE/Dazzle™ 594 anti-human CD86 (5 μl/test, 305433, BioLegend), and PE anti-human CD206 (5 μl/test, 321105, BioLegend) and incubated at 4 °C for 60 min. Wash the cells 2 ~ 3 times, add 500 μL cell staining buffer to resuspend the cells, and detected with FACSAria™ III (BD Biosciences CA, USA).

### Activators and inhibitors

Recombinant human IL-6 protein: (Sino Biological, 10395-HNAE, Beijing, China), recombinant mouse IL-6 protein: (Sino Biological, 50136-MNAE, Beijing, China), IL-6 neutralizing antibody: (Sino Biological, 10395-R508, Beijing, China), recombinant IgG protein (Sino Biological, 11047-TNAH, Beijing, China). JAK2 inhibitor, AZD-1480 5 μM (TargetMol, T3069, MA, USA). JAK2/STAT3 inhibitor, WP1066 10 μM (TargetMol, T2156, MA, USA).

### Western blotting

Protein expression was analyzed by using the western blot assay [27]. The following antibodies were used to assay: anti-JAK2 (Cell Signaling Technology, 3230, 1:1000), anti-pJAK2 (phosphor Y1007 + Y1008) (Cell Signaling Technology, 3771, 1:1000), anti-STAT1 (Cell Signaling Technology, 9172, 1:1000), anti-pSTAT1 (phosphor T701) (Cell Signaling Technology, 7649, 1:1000), anti-AKT (Cell Signaling Technology, 4685, 1:1000), anti-pAKT (phosphor S473) (Cell Signaling Technology, 4060, 1:1000), anti-ERK (Cell Signaling Technology, 4695, 1:1000), anti-pERK (phosphor T202 + T204) (Cell Signaling Technology, 4370, 1:2000), anti-STAT3 (Cell Signaling Technology, 12,640, 1:1000), anti-pSTAT3 (phosphor Y705) (Cell Signaling Technology, 9145, 1:2000), anti-C/EBPβ (Cell Signaling Technology, 90081, 1:1000), anti-IL6 (Absin, abs135607, 1:1000), anti-E-Cadherin (Abways, CY1155, 1:5000), anti-N-Cadherin (Abways, CY5015, 1:2000), anti-Vimentin (Abways, CY1155, 1:2000), anti-Snail (Cell Signaling Technology, 3879, 1:1000), anti-β-Catenin (Cell Signaling Technology, 8480, 1:1000), anti-GAPDH (Beyotime, AF0006, 1:5000), anti-β-ACTIN (Beyotime, AF0003, 1:5000).

### Plasmid, siRNAs constructs and transfections

The STAT3 (NM_003150, NM_213660) plasmid vector and C/EBPβ (NM_005194, NM_009883) plasmid vector were chemically synthesized, constructed, sequenced, and identified by Shanghai GeneChem Chemical Technology, Co. Ltd., China. STAT3-siRNA (NM_003150, NM_213660), C/EBPβ-siRNA (NM_005194, NM_009883), or negative control RNA (si-control) were chemically synthesized, constructed, sequenced, and identified by RiboBio Co. Ltd., Guangzhou, China. Short hairpin RNAs targeting IL-6 (NM_000600) and IL-6 overexpression plasmid ligated to a lentiviral vector were also chemically synthesized, constructed, sequenced, and identified by Shanghai GeneChem Chemical Technology, Co. Ltd., China. All plasmids (1 μg/μl) and siRNAs (20 μM) were transfected in macrophages using Advanced DNA RNA Transfection Reagent™ (ZETA LIFE, AD600025, CA, USA) following the manufacturer’s instructions. When the cells reached 60-80% confluence, the plasmid (12 μl) or siRNA (12 μl) was mixed 1:1 with the transfection reagent (12 μl), incubated at room temperature for 10-15 minutes, and added the complex to the cell culture plate, mixed gently. mRNA expression was detected after 48 hours and protein expression after 72 hours. Two different short hairpin RNAs targeting IL-6 (sh-IL6-1: GAACTTATGTTGTTCTCTA, sh-IL6-2: AGAACGAATTGACAAACAA) and an IL-6 overexpression plasmid were ligated to a lentiviral vector with puromycin resistance, transfected with HiTransG A (Genechem, Shanghai, China), changed the fluid after 12 hours, and puromycin was subsequently used to screen the cell lines.

### Chromatin immunoprecipitation (ChIP) assay

ChIP assays were performed using a SimpleChIP® Plus Enzymatic Chromatin IP Kit (Cell Signaling Technology, #9005, USA) according to the manufacturer’s instructions. The following antibodies were used to assay: STAT3 (Cell Signaling Technology, 12640, 1:50), C/EBPβ (Santa Cruz, sc-7962X, 1:50). The primer sequences were provided in Supplementary materials. The PCR products were analyzed by electrophoresis on the 1% agarose gel and detected through 4S red plus nucleic acid staining (Sangon Biotech, A606695, Shanghai, China).

### Luciferase reporter assay

DNA fragments with C/EBPβ or IL-6 transcription start regions (− 2000 bp - + 50 bp) or the mutant constructs of binding sites DNA fragments with firefly luciferase were cloned onto the pGL3-Basic plasmid (Genechem, Shanghai, China). Overexpression plasmids or empty plasmids and pGL3-Basic luciferase reporters were transfected into 293 T cells using Lipofectamine 3000 (Invitrogen, CA, USA), then treated with or without IL-6. Renilla luciferase reporter vector pRL-SV40 (Genechem, Shanghai, China) was provided as an internal transfection control. Luciferase activity was measured using a dual luciferase reporter system (Yeasen, 11402ES80, Shanghai, China). The intensity of firefly fluorescence and renilla fluorescence was measured at 560 nm and 450 nm, respectively.

### Immunohistochemistry

Immunohistochemistry experiments were performed according to our previous article [27]. The following antibodies were used to assay: E-cadherin (Abways, CY1155, 1:200), N-cadherin (Abways, CY5015, 1:200), Vimentin (Abways, CY1155, 1:200), IL-6 (Absin, abs135607, 1:200), CD206(Abcam, ab64693, 1:200).

### Animal experiments

All animal experiments were approved by the ethical committee of Zhongshan Hospital, Fudan University. For subcutaneous tumor formation experiments, 6 - 8 weeks of nude mice (BALB/c-nu) were randomly divided into five groups (*n* = 6 per group). 200 μl A549 cells (5 × 10^5^) or a mixture of A549 cells (5 × 10^5^) and TAMs (5 × 10^4^) were injected into the flank of the mice. After 10 days, the tumor size was measured every 5 days and the volume was calculated according to the following formula: Volume = 1/2 × (width^2^ × length). Mice were sacrificed after 30 days and tumor volume and weight were measured. Tumor tissues from mice were used for subsequent experiments, like WB and IHC.

### Statistics analysis

All statistical analyses were performed by R software 4.0.0, SPSS software 25.0, and Graphpad Software 9.0. Data are presented as the mean ± standard error of the mean (SEM). Student’s t-test was used to compare the differences between the two groups. Correlations between two variations were analyzed by Pearson’s correlation. Kaplan-Meier survival curves and log-rank tests were employed to depict overall survival (OS). The differences in tumor volume among groups were analyzed by analysis of variance (ANOVA), followed by a two-tailed Student’s t-test. The statistical significances of groups are represented as *p < 0.05, ** *p* < 0.01, *** *p* < 0.001, and **** *p* < 0.0001.

## Results

### TAMs in the TME are correlated with tumor progression and poor prognosis in LUAD patients

**Fig. 1:**
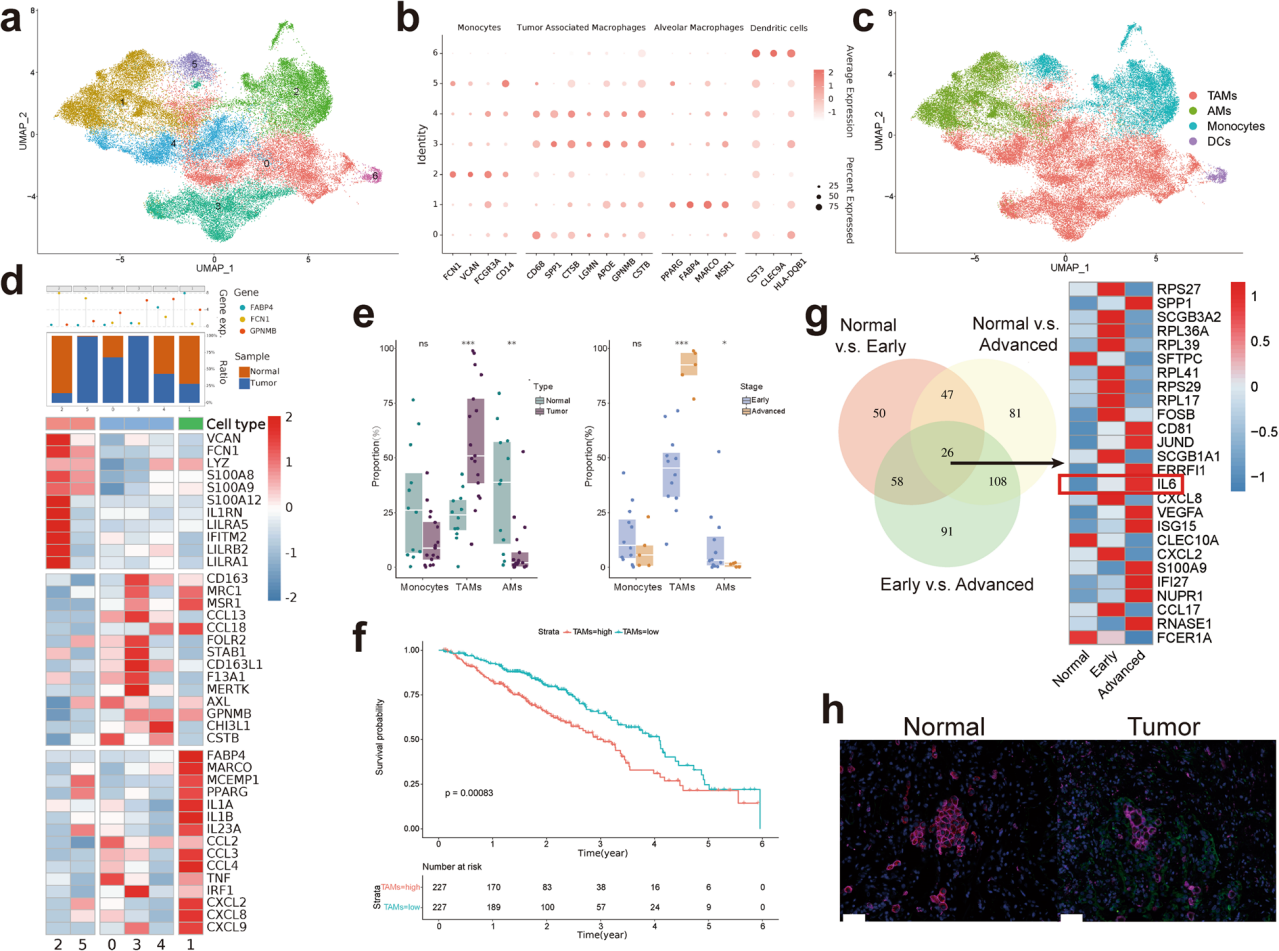
Annotation and function of myeloid cells and macrophages by scRNA-seq in tumor and normal tissue. **a** UMAP plot colored by different clusters. **b** Dot plot of mean expression of myeloid cells and macrophages’ characteristic genes in different clusters. **c** UMAP plot colored by different cell types. **d** Complex heatmap of selected marker genes in each cell cluster. Up: Tissue preference of each cluster. Down: Relative expression of marker genes associated with each cell subset. **e** Relative contribution of each cell type in normal vs. tumor tissue and early vs. advanced LUAD. **f** Kaplan–Meier curves of survival analysis in TCGA LUAD patients based on the TAMs infiltration. **g** Left: Difference genes between normal tissue, early LUAD, and advanced LUAD, with a threshold of |log2FC| > 0.5 & p_val_adj < 0.05, and take the intersection of the differential genes of the three groups. Right: Heatmap of 26 genes that differ among the three groups. **h** Immunofluorescence shows the expression of TAMs marker and IL-6 (Red CD68, Pink CD163, Green IL-6, Blue Dapi), scale bar represents 50 μm

To investigate the clinical significance of TAMs in LUAD, single-cell RNA sequencing data from 12 early LUAD patients, 5 advanced LUAD patients, and 12 normal lung tissues were used for analysis (Fig. 1a & Fig. S1a & b). Based on the marker genes of each subgroup, we divided myeloid cells into 7 clusters; Cluster 2 and 5 were monocytes; Cluster 6 was dendritic cells; Clusters 1 was defined as alveolar macrophages; and Clusters 0, 3, and 4 were defined as tumor-associated macrophages (TAMs) (Fig. 1b & c). In addition, the high-expression genes in each subpopulation also showed that monocytes mainly expressed classical monocyte-related genes, such as IL1RN and S100A8/9. TAMs mainly expressed anti-inflammatory factors such as CCL13 and FLOR2 and oncogenes such as SPP1, GPNMB, and CSTB. Alveolar macrophages are dominated by proinflammatory genes as well as scavenger receptors such as MARCO and MSR1 (Fig. 1d).

We found that TAMs infiltrated tumor tissues more than they did in normal tissues and that the proportion of TAMs increased with tumor progression (Fig. 1e). Subsequently, we used the deconvolution algorithm to map the scRNA-seq data to the TCGA database. Similarly, we found that the infiltration of TAMs gradually increased with tumor progression (Fig. S1c) and that the greater the infiltration of TAMs was, the worse the prognosis of patients was (Fig. 1f).

Thereafter, we compared the differential genes of TAMs in normal tissue, early LUAD tissue, and the advanced LUAD TME, and found that IL-6 secretion was significantly different among the three groups, increasing with tumor progression (Fig.1g). And in our scRNA data, we similarly found that IL-6 was secreted by myeloid cells, especially within tumor tissue (Fig. S1d). Therefore, we investigated the correlation between TAMs and IL-6. The results showed a significant correlation between the TAM-specific markers CD68 and CD163 with IL-6, which was similarly demonstrated by our mIHC data (Fig. 1h & Fig. S1e). These data suggest that TAMs in the TME may promote tumor progression through the secretion of IL-6, which in turn affects patient prognosis.

### IL-6 promotes lung adenocarcinoma progression by activating TAMs and is associated with a worse prognosis

**Fig. 2:**
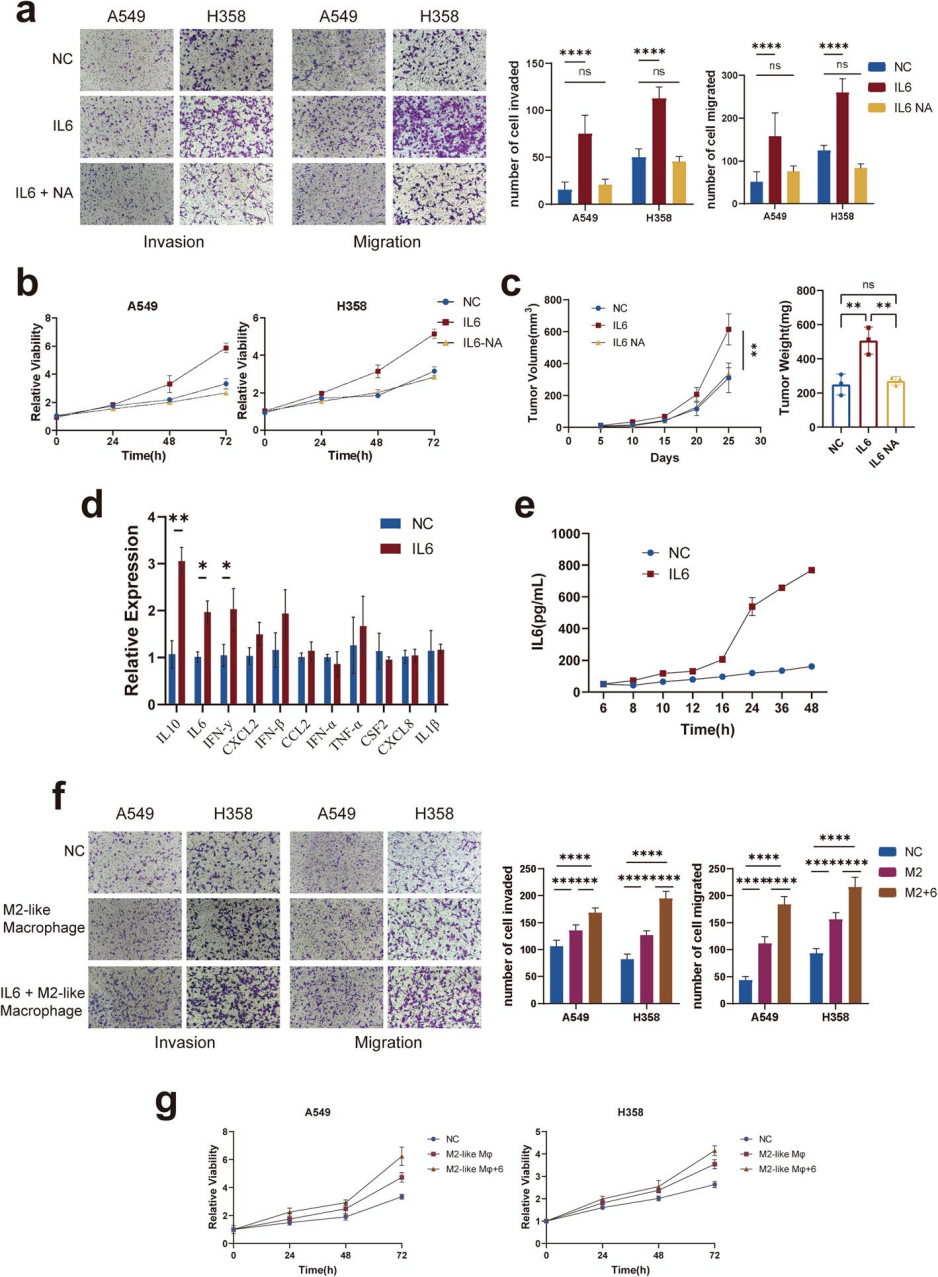
TAMs promote LUAD cell proliferation, migration and invasion by secreting IL-6. **a** Cell migration and invasion ability of LUAD cells (A549 and H358) alone, stimulated with IL-6 or stimulated with IL-6 followed by addition of IL-6 neutralizing antibody was determined by the transwell assay. **b** Cell proliferation ability of LUAD cells (A549 and H358) alone, stimulated with IL-6 or stimulated with IL-6 followed by addition of IL-6 neutralizing antibody was determined by the CCK8 assay. **c** The tumor size and tumor weight in the A549 alone, A549 + IL-6 stimulation and A549 + IL-6 stimulation + IL-6-NA groups. **d** RT-qPCR analyzed the expression of the most often released cytokines by macrophages after IL-6 stimulation. **e** Elisa analysis of IL-6 showed changes in macrophage IL-6 expression over time after IL-6 stimulation. **f** & **g** Cell migration, invasion and proliferation ability of LUAD cells (A549 and H358) alone, co-culture with M2-like macrophages or co-culture with M2-like macrophages and stimulated with IL-6 was determined by the transwell assay and CCK8 assay

To determine the role of IL-6 in LUAD development, we first used the TCGA database to compare the relationship between IL-6 expression and prognosis. We found that high IL-6 expression was related to worse overall survival (OS) in lung cancer patients (*P* = 0.0032) (Fig. S2a). In addition, to test whether IL-6 can promote the progression of lung adenocarcinoma, we used IL-6 to stimulate two different LUAD cell lines, A549 and H358. We observed that the proliferation, invasion, and migration abilities of LUAD cells were greatly enhanced. In contrast, the malignant phenotype of LUAD cells was comparable to that of the control group after the addition of neutralizing antibodies (Fig. 2a & b). Similarly, in vivo experiments revealed that exogenous IL-6 promoted the proliferation of A549 cells in nude mice, while the addition of an IL-6 neutralizing antibody significantly inhibited this effect (Fig. 2c & Fig. S2b). These results illustrate that IL-6 can promote malignant phenotypes in LUAD cells and that exogenous IL-6 is the main cause of these changes.

To further elucidate the role of IL-6 in the TME, we assessed the expression of IL-6 receptors in different cell subtypes by using scRNA-seq data. Interestingly, the results showed that the IL-6 receptors IL6R and GP130 were highly expressed not only in tumor cells but also in TAMs (Fig. S2c). We used RT-qPCR to determine the cytokines most often released by macrophages after IL-6 activation. Surprisingly, in addition to the expression of proinflammatory chemokines such as IL-10, the mRNA expression of IL-6 was also increased after stimulation (Fig. 2d & Fig. S2d). Subsequently, macrophages were stimulated with IL-6 and the medium was changed after 4 h. IL-6 secreted by macrophages was measured via Elisa. Similarly, we found an increase in IL-6 secretion (Fig. 2e). Given that macrophages play a crucial role via the secretion of various cytokines in the TME and that both the IL-6 and IL-6 receptors are highly expressed by TAMs, we speculate that there is a positive feedback loop involving IL-6 in TAMs, where IL-6 stimulates increased IL-6 secretion from TAMs and promotes LUAD progression.

Subsequently, to investigate the influence of IL-6 stimulated TAMs on LUAD, we co-cultured the M2-like macrophages induced by THP1 cells with LUAD cells (Fig. S3a & b). To prevent the effect of exogenous IL-6 on LUAD cells, we first stimulated M2-like macrophages with IL-6 for 4 h, followed by a medium change, and then co-cultured them with LUAD cells. The addition of IL-6 to M2-like macrophages significantly promoted the proliferation, invasion, and migration ability of lung adenocarcinoma cells (Fig. 2f & g). Taken together, these findings demonstrated that TAMs promote tumor progression may by enhancing IL-6 secretion through IL-6.

### IL-6 can activate the JAK2/STAT3 pathway in M2-like macrophages and promote LUAD progression

**Fig. 3:**
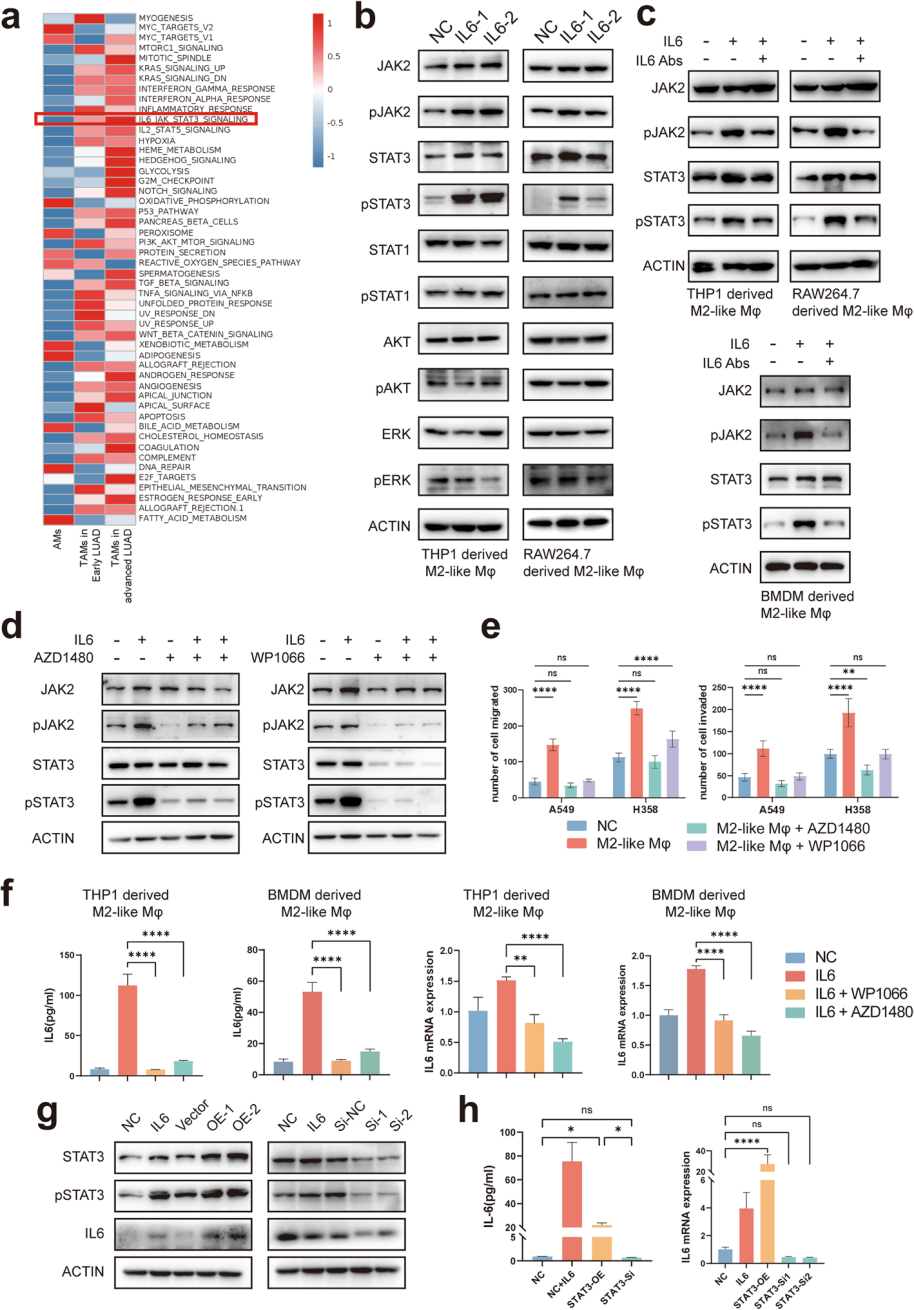
IL-6 promotes LUAD progression by activating the JAK2/STAT3 pathway in M2-like macrophages. **a** GSVA analysis of differential pathways between AMs, TAMs in early and advanced LUAd. **b** Effects of IL-6 stimulation on common pathway expression in TAMs detected by western blot analysis. **c** Expression of the JAK2/STAT3 pathway with IL-6-stimulated, IL-6-stimulated with the addition of IL-6-NA in M2-like macrophages were analyzed by western blot. **d** Effect of AZD1480 and WP1066 on JAK2/STAT3 pathway expression in THP1 derived M2-like macrophages after IL-6 stimulation were analyzed by western blot. **e** Cell migration and invasion ability of LUAD cells alone, co-culture with M2-like macrophages or co-culture with M2-like macrophages followed by addition of AZD1480 and WP1066 was determined by the transwell assay. **f** RT-qPCR and Elisa analysis showed IL-6 expression in M2-like macrophages after IL-6 stimulation or IL-6 stimulation with the addition of AZD1480 and WP1066 (Left: Elisa results. Right: RT-qPCR results). **g** IL-6 expression after STAT3 overexpression or knockdown was analyzed by western blot. **h** RT-qPCR and Elisa analysis showed IL-6 expression in M2-like macrophages after STAT3 overexpression or knockdown

To clarify the mechanism of ‘the impact of IL-6 on TAMs, we performed GSVA analysis on single-cell data and found that the downstream JAK/STAT3 pathway of IL-6 was significantly activated and further increased with tumor progression (Fig. 3a). We then examined the expression of common downstream pathways of IL-6 in M2-like macrophages after IL-6 stimulation. In addition to THP1-derived M2-like macrophages, we further validated these findings with mouse BMDM derived M2-like macrophages and RAW 264.7-derived M2-like macrophages (Fig. S3c & d). The results showed that activation of the JAK2/STAT3 pathway was most pronounced (Fig. 3b), whereas the treatment with the IL-6 neutralizing antibody inhibited the induction of pJAK2 and pSTAT3 expression (Fig. 3c). JAK2/STAT3 is a classical IL-6 signaling pathway that can promote inflammation and tumor progression. IL-6 first activates JAK2 in the cell membrane, which in turn phosphorylates STAT3, then pSTAT3 returns to the nucleus and acts as a transcription factor to boost downstream gene expression [28]. After that, we added two JAK2/STAT3 pathway inhibitors (AZD1480 and WP1066) to M2-like macrophages after 4 h of IL-6 stimulation (Fig. S4a) and subsequently co-cultured them with LUAD cells to determine whether IL-6 promoted LUAD progression through the JAK2/STAT3 pathway. The aforementioned two inhibitors strongly suppressed JAK2 and STAT3 expression (Fig. 3d and Fig. S4b) and eliminated the promotion of LUAD by TAMs after IL-6 stimulation (Fig. 3e and Fig. S4c). However, both inhibitors had slight inhibitory effects on the invasion and migration ability of LUAD cells, but the effects were not different (Fig. S4e).

Thus, these data indicate that IL-6-stimulated TAMs may promote lung adenocarcinoma progression through activation of the JAK2/STAT3 pathway.

### STAT3 can regulate IL-6 expression in TAMs

To test whether STAT3 is essential for the expression of IL-6, we first examined the effects of the aforementioned two JAK/STAT3 pathway inhibitors on IL-6 expression. The results revealed that these compounds were able to inhibit the transcription and expression of IL-6 (Fig. 3f). Subsequently, we transfected M2-like macrophages with a STAT3 overexpression vector (STAT3-OE) or STAT3 siRNA (si-STAT3) to overexpress or knock down STAT3, respectively (Fig. S5a). After validating the overexpression and knockdown effects, we found that the change in IL-6 expression was consistent with the trend of pSTAT3 change, as determined by western blot analysis (Fig. 3g). Moreover, RT-qPCR and Elisa results also revealed that the transcriptional and translational changes in IL-6 were consistent with the alteration of pSTAT3 (Fig. 3h). Next, we utilized the chromatin immunoprecipitation test (ChIP) assay to determine whether STAT3 can bind to the IL-6 transcriptional start site and thus influence IL-6 expression as a transcription factor (Supplementary Table 1). However, our results showed that STAT3 is unable to bind to the possible binding site in the IL-6 transcriptional start region (Fig. S5b).

To further explore how STAT3 can regulate IL-6 expression, we used JASPAR to analyze the transcription factors that may regulate IL-6 and may be regulated by STAT3. After taking the intersection, the C/EBPβ is found to be satisfactory. Therefore, we hypothesized that STAT3 regulates C/EBPβ transcription, which in turn may regulate IL-6 expression.

### C/EBPβ plays a crucial role in LUAD progression and may be regulated by pSTAT3

**Fig. 4:**
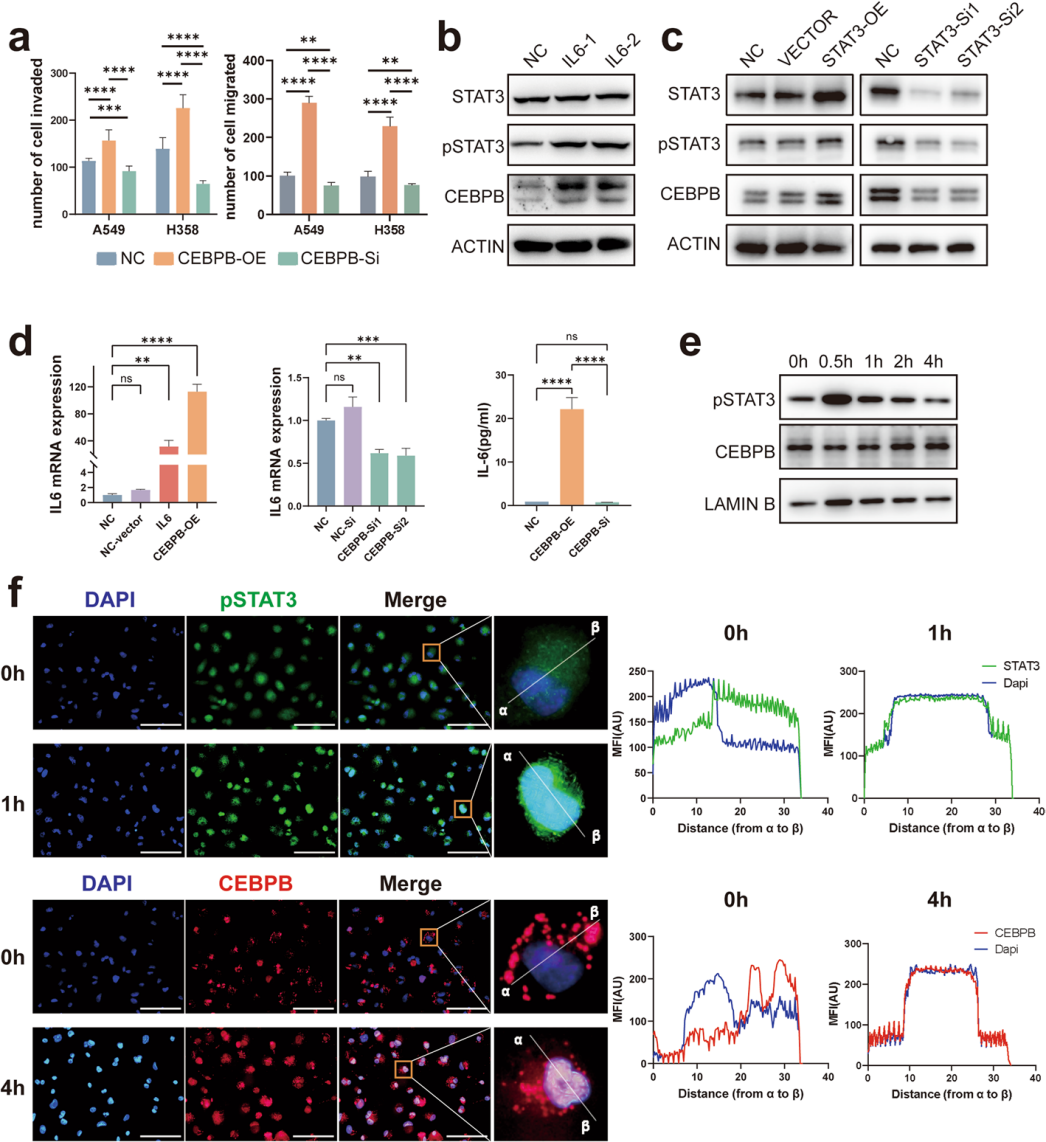
pSTAT3 promotes tumor progression by regulating C/EBPβ expression in TAMs. **a** Cell migration and invasion ability of LUAD cells, C/EBPβ overexpression or C/EBPβ knockdown was determined by the transwell assay. **b** Effects of IL-6 stimulation on C/EBPβ expression in TAMs. **c** Expression of C/EBPβ after STAT3 overexpression or knockdown. **d** RT-qPCR and Elisa analysis showed IL-6 expression in M2-like macrophages after C/EBPβ overexpression or knockdown. **e** Expression of pSTAT3 and C/EBPβ in the nucleus after IL-6 stimulation. **f** The localization of pSTAT3 and C/EBPβ were analyzed by immunofluorescence staining. The nuclei were stained with 4′,6-diamidino-2-phenylindole (DAPI; blue). The line charts represent fluorescence intensity (MFI), presenting the distance from α to β, scale bar represents 100 μm

We subsequently sought to determine the crucial role of C/EBPβ in LUAD progression. By using the TCGA database, we discovered that patients with high C/EBPβ expression had significantly worse OS and recurrence-free survival (RFS) (*P* < 0.05) (Fig. S6a & b). Then, we overexpressed or knocked down C/EBPβ in M2-like macrophages (Fig. S6c), and subsequently co-cultured these macrophages with LUAD cell lines. We found that in the C/EBPβ-OE group, the invasion and migration ability of LUAD cells were significantly enhanced, while in the C/EBPβ-KD group, these changes were significantly eliminated (Fig. 4a). Overall, the results obtained in our study suggest that C/EBPβ plays an important role in the progression of LUAD.

To explore the correlation between STAT3- C/EBPβ-IL-6 in TAMs, we first examined the expression of C/EBPβ at the mRNA and protein levels after IL-6 stimulation. The expression of C/EBPβ, particularly the transcription-promoting isoform LAP, was significantly upregulated after stimulation and earlier than that of IL-6, whereas the expression of the inhibitory isoform LIP was not significantly changed (Fig. 4b & Fig. S6d & e). We also found a correlation between IL-6 and C/EBPβ expression in the TCGA database (Fig. S6f). In addition, overexpression or knockdown of STAT3 resulted in similar changes in C/EBPβ expression (Fig. 4c). Additionally, we observed an increase in IL-6 transcription and expression in cells overexpressing C/EBPβ, while the opposite effect was observed when C/EBPβ was knocked down (Fig. 4d). Considering that pSTAT3 and C/EBPβ operate as transcription factors in the nucleus, we examined the expression of pSTAT3 and C/EBPβ in the nucleus following IL-6 stimulation. We found that pSTAT3 expression in the nucleus peaked only half an hour after IL-6 stimulation and then gradually decreased, whereas C/EBPβ expression peaked more than 4 h after stimulation (Fig. 4e). In addition, our immunofluorescence results also showed that pSTAT3 was significantly nuclearized after half an hour of IL-6 stimulation. However, 4 hours after stimulation, the entry of C/EBPβ into the nucleus peaked (Fig. 4f). This finding supports the idea that C/EBPβ can be temporally regulated by pSTAT3. We validated that C/EBPβ is involved in IL-6 mediated lung carcinogenesis of TAMs and that it is potentially regulated by pSTAT3.

### STAT3-C/EBPβ-IL6 signaling is responsible for IL-6 induced cancer progression

**Fig. 5:**
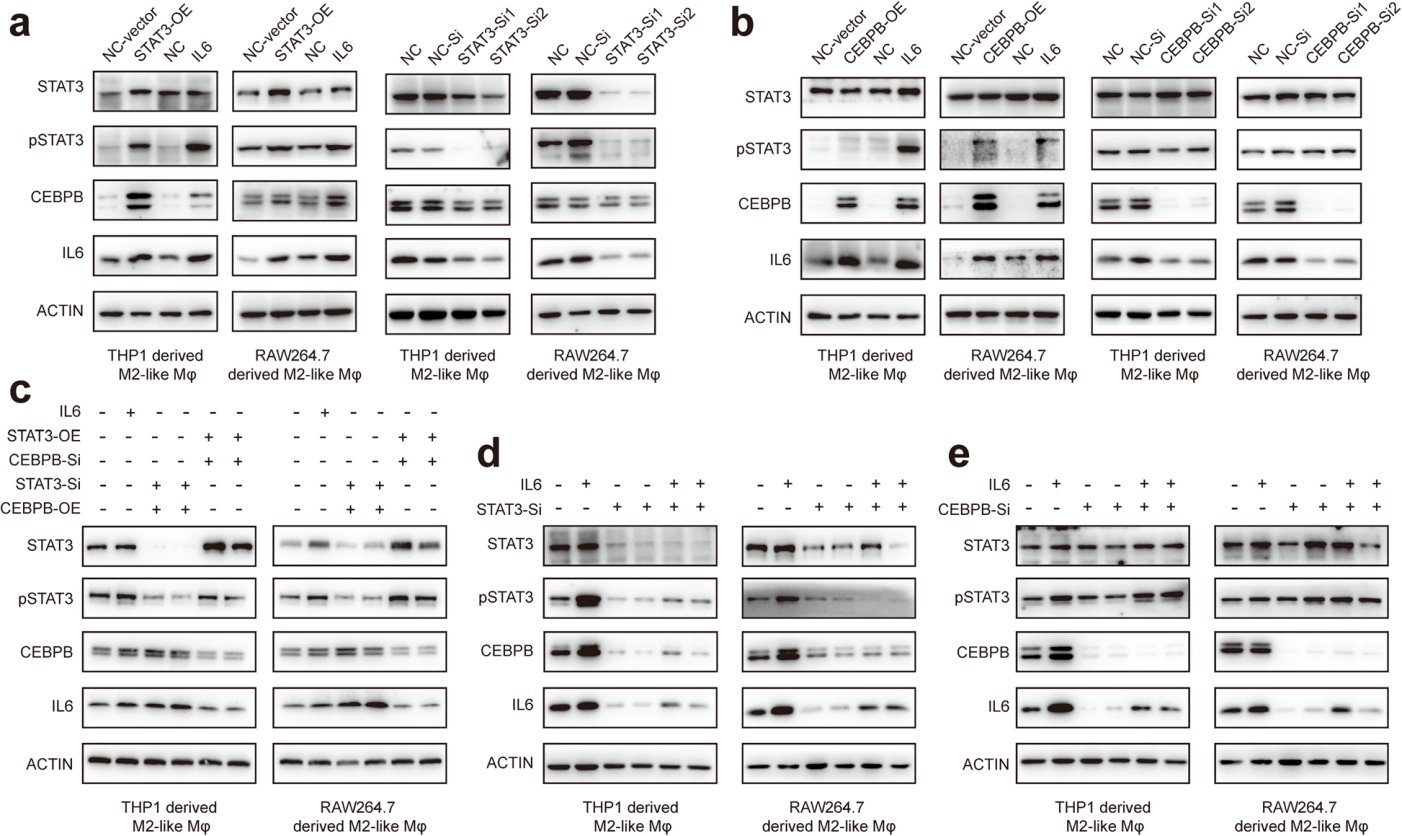
Regulatory role of STAT3-C/EBPβ-IL6. **a** C/EBPβ and IL-6 expression after STAT3 knockdown or overexpression. **b** STAT3 and IL-6 expression after C/EBPβ knockdown or overexpression. **c** IL-6 expression after STAT3 knockdown and C/EBPβ overexpression or STAT3 overexpression and C/EBPβ knockdown. **d** IL-6 expression after STAT3 knockdown and rescued by IL-6 stimulation. **e** IL-6 expression after C/EBPβ knockdown and rescued by IL-6 stimulation

To investigate whether pSTAT3 can regulate C/EBPβ and consequently the expression of IL-6, we determined how the pSTAT3-C/EBPβ-IL6 axis was expressed in two different M2-like macrophage cell lines after activation or inhibition of STAT3 or C/EBPβ. C/EBPβ and IL-6 expression was shown to be strongly elevated following STAT3 overexpression, but decreased after STAT3 knockdown (Fig. 5a). We observed similar alterations in IL-6 expression following C/EBPβ knockdown or overexpression (Fig. 5b). However, there was no substantial change in IL-6 expression after STAT3 overexpression combined with C/EBPβ knockdown (Fig. 5c). In contrast, IL-6 stimulation after STAT3 or C/EBPβ knockdown did not significantly increase IL-6 expression in M2-like macrophages (Fig. 5d & e). Using the aforementioned overexpression, knockdown, and reversion assays, we preliminarily confirmed the regulatory effects of pSTAT3 on C/EBPβ and C/EBPβ on IL-6.

**Fig. 6:**
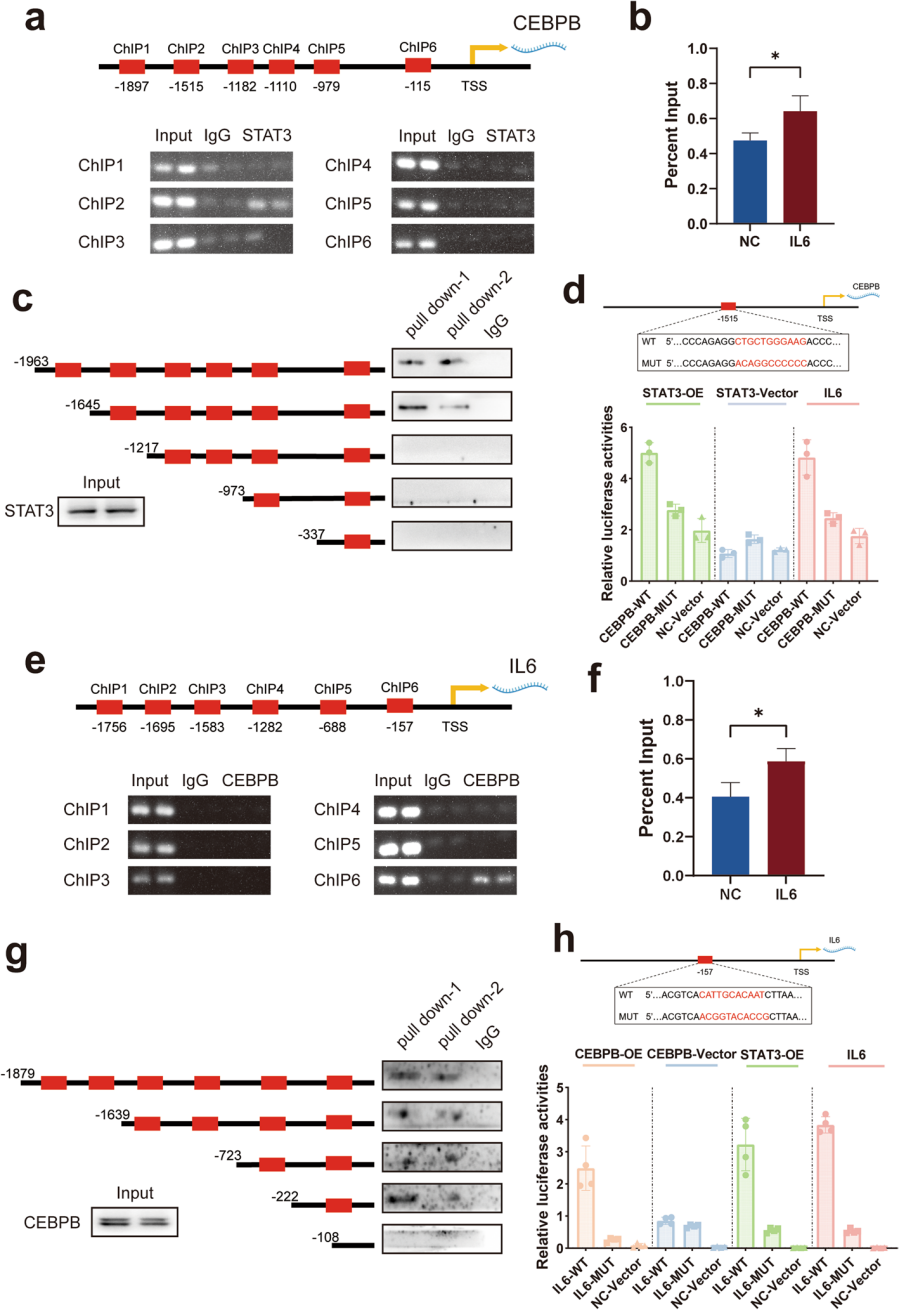
Mechanisms of STAT3 regulation of C/EBPβ and C/EBPβ regulation of IL-6. **a** ChIP assay demonstrated the binding of STAT3 to the C/EBPβ promoter in THP1-derived M2-like macrophages. **b** RT-qPCR of the ChIP products confirmed the binding capacity of STAT3 to the C/EBPβ promoter. **c** Selective mutation analyses identified STAT3-responsive regions in the C/EBPβ promoter. **d** Luciferase reporter plasmids with wild and mutated C/EBPβ promoters were transfected into 293 T cells. Relative luciferase activity was determined using IL-6 stimulation or STAT3 overexpression. **e** ChIP assay demonstrated the binding of C/EBPβ to the IL-6 promoter in. **f** RT-qPCR of the ChIP products confirmed the binding capacity of C/EBPβ to the IL-6 promoter. **g** Selective mutation analyses identified C/EBPβ-responsive regions in the IL-6 promoter. **h** Luciferase reporter plasmids with wild and mutated IL-6 promoters were transfected into 293 T cells. Relative luciferase activity was determined using IL-6 stimulation, C/EBPβ or STAT3 overexpression

In the following, to further demonstrates the specific regulatory mechanism of pSTAT3 on C/EBPβ and of C/EBPβ on IL-6. First, we used the JASPAR database to predict possible transcription factor binding sites for STAT3 on C/EBPβ (Supplementary Table 2). We designed primer sets containing putative STAT3 binding sites to amplify the portion of the C/EBPβ promoter. ChIP revealed that pSTAT3 was able to bind to the C/EBPβ transcription start region at the − 1645 to − 1358 site, whereas the remaining region was not regulated by STAT3. Among them, the − 1515 to − 1505 site is one of the potential binding sites we predicted to exist (Fig. 6a). In addition, the binding capability dramatically increased after IL-6 stimulation (Fig. 6b). Subsequently, we generated a series of 5′ deletion constructs of the C/EBPβ promoter and determined whether STAT3 was able to bind to this segment using DNA pull-down experiments. The results also suggested that the region between the − 1645 a–d - 1217 sites is responsible for the STAT3-mediated regulation of C/EBPβ (Fig. 6c). To further confirm this interaction, a dual-luciferase reporter assay was performed. We designed wild-type and − 1515 to − 1505 site mutant C/EBPβ transcription start region luciferase reporter plasmids and transfected them into 293 T cells. The results demonstrated that the transcriptional capacity of C/EBPβ was significantly enhanced after IL-6 stimulation or STAT3 overexpression, while this capacity was significantly diminished after mutation of the binding site (Fig. 6d).

The regulatory effect of C/EBPβ on IL-6 was subsequently be confirmed. First, we predicted several binding sites using the JASPAR database (Supplementary Table 3). Similarly, we used a ChIP assay to confirm that C/EBPβ can bind to the IL-6 transcriptional start region at − 157 to − 147 bp (Fig. 6e). The ChIP-PCR results showed that the binding capability increased after IL-6 stimulation (Fig. 6f). DNA pull-down assay also confirmed the regulatory region of C/EBPβ on IL-6 is located at − 157 to − 147 bp (Fig. 6g). Next, a dual-luciferase reporter assay revealed that IL-6 stimulation or STAT3 or C/EBPβ overexpression increased IL-6 transcription, whereas mutation of the binding site reduced IL-6 transcription (Fig. 6h). As a result of these the previous studies, we concluded that STAT3 enhances C/EBPβ transcription and thus IL-6 expression after IL-6 stimulation in TAMs.

### IL-6 promotes LUAD cell migration and invasion through activation of the EMT pathway in vivo and in vitro

**Fig. 7:**
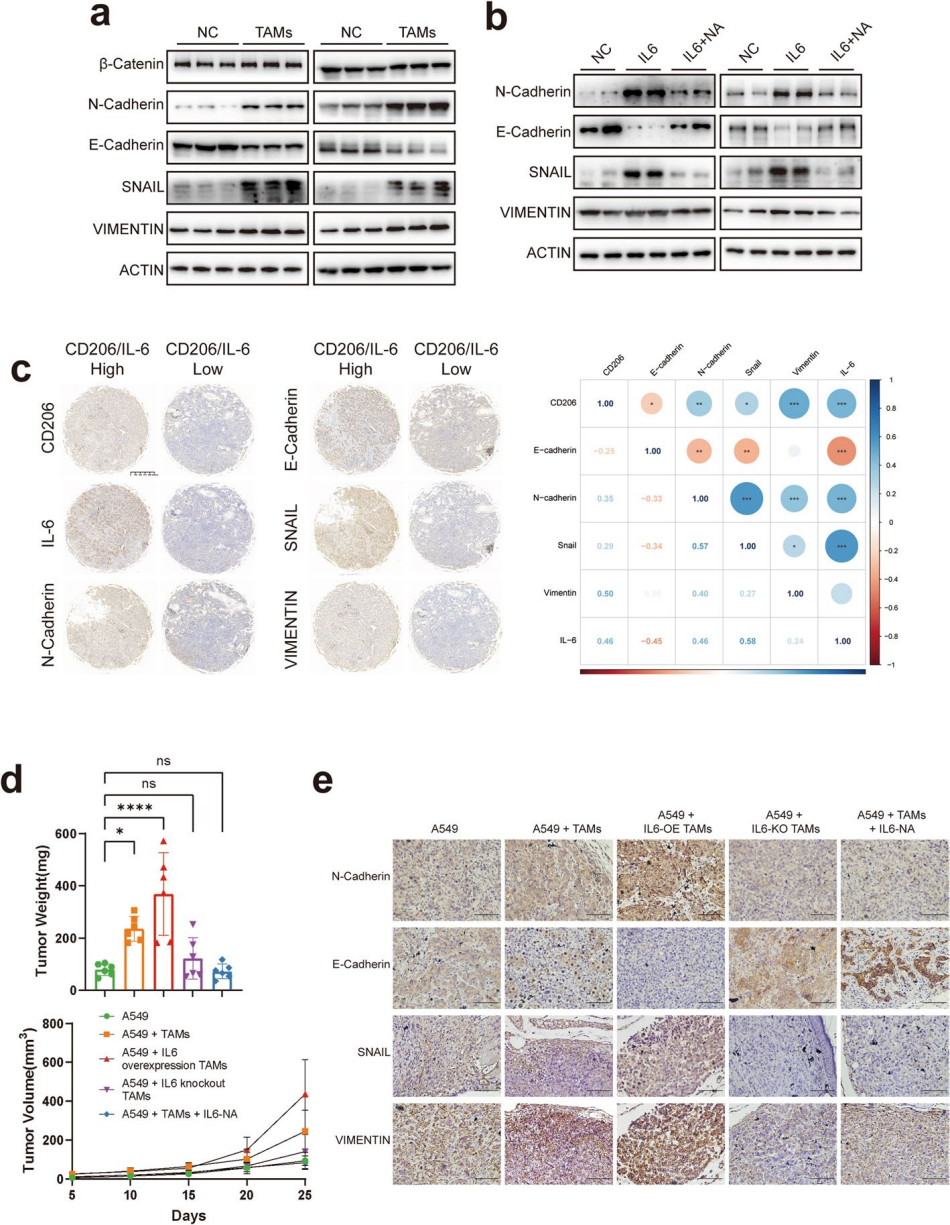
IL-6 promotes LUAD migration and invasion through activation of EMT pathway in vivo and in vitro. **a** Expression of EMT-related genes after co-culture with M2-like macrophages. **b** Expression of EMT-related genes using IL-6 stimulation or IL-6-neutralising antibodies. **c** IHC analyzed the expression of CD206, IL-6, E-cadherin, N-cadherin, Vimentin and Snail, and their relationships of tumors from LUAD patients. **d** The tumor size and tumor weight in the A549 alone, A549 + control TAMs, A549 + IL-6 overexpression TAMs, A549 + IL-6 knockout TAMs, and A549 + TAMs + IL-6 neutralizing antibodies groups. **e** IHC analyzed the expression of E-cadherin, N-cadherin, Snail and Vimentin protein of tumors from the subgroups above. Scale bar represents 10 μm

As M2-like macrophages significantly promoted LUAD invasion and migration by secreting IL-6, we investigated gene alterations related to invasion and migration. After detecting the expression of EMT and metalloproteinase-related genes in lung adenocarcinoma cells co-cultured with M2-like macrophages, we found that the expression of EMT-related genes (E-cadherin, N-cadherin, Snail, Vimentin) was highly variable (Fig. S7a). Next, we examined the expression of EMT-related genes in LUAD cells co-culture with M2-like macrophages and found that the expression of E-cadherin was decreased, while the expression of N-cadherin and Snail was increased (Fig. 7a). Co-culture with M2-like macrophages also partially promotes the EMT pathway in the non-tumorigenic lung epithelial cell line BEAS-2B, but the activation is not as pronounced as in tumor cells (Fig. S7b). The TCGA database also showed a correlation between TAM markers and EMT-related genes in LUAD patient samples (Fig. S7c). Subsequently, we used IL-6 to directly stimulate LUAD cells and found the same changes as those observed after co-culture with M2-like macrophages, whereas these changes were eliminated by the addition of IL-6 neutralizing antibodies (Fig. 7b). In addition, we collected LUAD samples from 64 patients who underwent surgery at Zhongshan Hospital, Fudan University between 2022.1 to 2022.12, and made a tissue chip. The expressions of TAMs markers, CD206, IL-6 and EMT were verified using immunohistochemistry anylisis. The results confirmed that the expression of TAMs markers was inversely correlated with E-cadherin and positively correlated with the expression of N-cadherin, Snail, and Vimentin (Fig. 7c).

To demonstrate the above in vitro results, an in vivo xenograft model was used. Consequently, A549 cells alone, A549 cells mixed with control TAMs, A549 cells mixed with IL-6 overexpressing TAMs, A549 cells mixed with IL-6 knockout TAMs, and A549 cells mixed with TAMs and IL-6 neutralizing antibodies were injected into the flanks of nude mice. M2-like macrophages were discovered to play a substantial role in the progression of lung adenocarcinoma, and this effect was enhanced by IL-6 overexpression. However, these effects were attenuated by IL-6 knockdown or by the use of an IL-6 neutralizing antibody (Fig. 7d & Fig. S7d). Immunohistochemical analysis results of tumor tissues also showed that the expression of EMT pathway components was significantly activated after IL-6 overexpression, and these effects were eliminated after IL-6 knockdown or treatment with an IL-6 neutralizing antibody (Fig. 7e). The results of this in vivo experiment suggested that TAMs promoted the EMT pathway in LUAD cells and promoted lung cancer progression through the secretion of IL-6.

## Discussion

Metastasis is the most important factor leading to tumor death in lung cancer patients. Although target therapy and immunotherapy have achieved impressive results for advanced lung cancer patients, the 5-year survival rate is only 20-30% [29]. Therefore, it is urgent to unravel the molecular mechanisms of NSCLC metastasis and provide new therapeutic strategies to improve patient prognosis. In our study, we found that a microenvironment with high IL-6 expression in lung cancer was able to activate the JAK2/STAT3 pathway in TAMs, and STAT3, a transcription factor, was able to promote C/EBPβ expression. Further studies revealed that C/EBPβ could activate IL-6 transcription, resulting in a positive feedback loop of IL6-JAK2/STAT3/C/EBPβ-IL6 in TAMs. IL-6 can further activate the EMT pathway in lung adenocarcinoma cells and promote metastasis, and the use of neutralizing antibodies against IL-6 can inhibit the metastasis of lung cancer in vitro and in vivo.

Given the heterogeneity of tumors, it is likely that only a small number of cells contribute to the invasiveness of lung cancer. Therefore, by analyzing these cells via single-cell RNA sequencing, we found that tumor-associated macrophages are closely associated with the progression and poorer prognosis of lung cancer [14]. Recently, growing evidence has suggested that TAMs are linked to tumor progression. Restle et al. reported that a suppressive tumor immune microenvironment (TIME) exists in patients with brain metastases from lung cancer and that TAMs are associated with this suppressive TIME [30]. Similarly, Kim et al. found that myeloid cells shape the immunosuppressive microenvironment of the tumor by single-cell RNA sequencing, while tumor metastases are enriched with more anti-inflammatory macrophage subtypes [31]. However, the mechanism by which TAMs promote lung cancer progression and metastasis remains controversial.

EMT is the transition of epithelial cells to mesenchymal cells. Activation of the EMT is a key process in cancer cell metastasis. During this process, epithelial cells acquire the characteristics of mesenchymal cells with increased motility and migration. It is characterized by a decrease or loss of expression of epithelial cell markers (E-cadherin and keratin) and an increase in the expression of mesenchymal cell markers (vimentin and N-cadherin) [32]. EMT causes epithelial cells to lose cell polarity, reorganize the cytoskeleton, and reprogram gene expression, promoting an aggressive phenotype during cancer metastasis [33]. Thus, EMT is an important mechanism in tumor metastasis. Previous studies have shown that vimentin and N-cadherin mRNA and protein are significantly greater in the tumor tissues of NSCLC patients than in neighboring tissues, whereas E-cadherin expression is significantly lower [34]. Our study is consistent with previous studies in which we found that TAMs can promote tumor progression and metastasis by activating EMT in tumor cells. Guo et al. reported that M2 macrophages promote malignancy in lung cancer through EMT by upregulating CRYAB expression and activating the ERK1/2/Fra-1/slug signaling pathway [17]. Li et al. demonstrated that CD68+ TAMs both reduced snail expression and inhibited tumor buds, which was negatively correlated with the EMT phenotype in colorectal cancer [35].

Considering that macrophages usually perform biological functions by secreting cytokines and chemokines. We found that IL-6 expression in TAMs gradually increased with tumor progression and played an important role in macrophage-mediated activation of EMT in lung adenocarcinoma. It was able to promote the progression, invasion, and metastasis of lung adenocarcinoma cells in vivo and in vitro. IL-6 is a multifunctional cytokine that mediates immune and inflammatory responses, and it is involved in promoting angiogenesis, tumorigenesis, and progression through complex mechanisms, such as increasing the expression of invasion-related genes (Twist and MMP-1) and antiapoptotic factors (Bcl-2 and Bcl-xL), and activation of the PI3K, ERK, and STAT3 signaling pathways [36, 37]. Previous studies have also shown that elevated IL-6 levels are associated with an increased risk of disease progression in patients with severe lung cancer, whereas other inflammatory cytokines are not significantly associated with disease progression [38]. Interestingly, in our study, the secretion of IL-6 by macrophages promoted the secretion of additional IL-6, which suggested that a positive feedback loop in TAMs promotes IL-6 secretion. In addition, IL-6 can activate the EMT pathway in lung adenocarcinoma and promote metastasis, whereas the use of an anti-IL-6 monoclonal antibody eliminated this effect. Thus, targeting IL-6 may be a potential pharmacological approach for treating lung cancer.

Furthermore, in TAMs, the activation of the JAK2/STAT3 pathway was the most pronounced downstream pathway after IL-6 stimulation. The JAK/STAT pathway is the classical signaling pathway for IL-6. Extracellular IL-6 binds to IL-6R on the membrane to form a complex, which induces gp130 to aggregate and bind to it, forming a heterohexamer consisting of two IL-6, two IL-6R, and two gp130 molecules, followed by the activation of the complex to activate JAK, which enters the nucleus through the activation of STAT3 to form a dimer and regulate the transcription of the target genes [28, 39]. Clinical studies have shown that STAT3 is persistently activated in 22-65% of NSCLC patients, and STAT3 activity is correlated with tumor progression, poor prognosis, and short patient survival [40]. Similarly, activation of the JAK2/STAT3 pathway in TAMs promoted lung cancer metastasis, which was attenuated by knockdown or the use of JAK2/STAT3 inhibitors. Previous studies have shown that, CAFs in the TME can secrete IL-6, which stimulates STAT3 signaling in lung cancer cells and promotes metastasis [8]. Bo Jing et al. showed that IL-6/STAT3 signaling-induced immunosuppression is essential for lung metastasis, and IL-6/STAT3 promotes lung metastasis not only through a cell-autonomous propensity for metastasis but also by establishing a metastatic niche [41]. Once the STAT complex enters the nucleus, it performs tasks related to transcriptional regulation, such as engagement of chromatinized target genes, binding response elements, and recruitment of transcriptional co-activators that recruit and activate RNA Pol II [42]. Multiple observations have been made concerning the interactions between STATs and other transcription factors, such as IRFs and the NFκB family, including the regulation of transcription factor synthesis, the regulation of transcription factor activity, and direct or indirect interactions within the genome that affect transcription [43]. In the present study, we found that IL-6 expression was regulated by STAT3, but this regulation was indirect and involved C/EBPβ as an intermediary. STAT3 binds directly to relevant sites in the C/EBPβ promoter region, thereby regulating the transcription of C/EBPβ, which in turn regulates IL-6 transcription and promotes IL-6 secretion in TAMs. C/EBPβ is a member of the CCAAT/enhancer binding protein (C/EBP) family of basic leucine zipper-class transcription factors, which have been implicated in various cellular functions, such as cellular energy metabolism, cell proliferation, and differentiation [44]. Our previous study showed that metformin inhibits the proliferation of lung cancer cells and exerts anti-tumor effects in an AMPK-C/EBPβ-PDL1 signaling-dependent manner [45]. In our study, both single-cell data and in vitro experiments revealed that C/EBPβ can regulate IL-6 expression, and we further clarified and demonstrated its potential regulatory sites. Our results demonstrated that TAM-tumor interactions are important regulators of the malignant phenotype of cancer cells. In addition, we have provided strong evidence that the JAK2/STAT3/C/EBPβ-induced IL-6 positive feedback pathway is important for lung cancer cell growth, migration, and invasion, which suggests that the STAT3-C/EBPβ-IL-6 signaling axis plays a crucial role in TAM-mediated lung cancer progression.

Nevertheless, our study also has some limitations, although we confirmed our results using single-cell technology and in vivo experiments, there is still a gap in the TAMs phenotypes between in vitro-induced and in tumors. Secondly, the efficacy of the investigated inhibitors must be further validated in the application of clinical trials.

## Conclusion

In conclusion, our current findings reveal an important mechanism that discloses the role of TAMs on cancer cells through the IL6-STAT3-C/EBPβ-IL6 positive feedback loop in the TME. And this mechanism is crucial for lung cancer development, progression, and metastasis. Our study highlights that the IL6-STAT3-C/EBPβ-IL6 signaling cascade may be a potential therapeutic target against lung cancer.

### Supplementary Information

**Supplementary material 1.**

## Data Availability

Single-cell data after integration are available upon request to corresponding authors.

## Abbreviations

LUAD: Lung adenocarcinoma
TAMs: Tumor-associated macrophages
TME: Tumor microenvironment
IL-6: Interleukin 6
STAT3: Signal transducer and activator of transcription 3
C/EBPβ: CCAAT enhancer binding protein beta
ChIP: Chromatinimmunoprecipitation
EMT: Epithelial-mesenchymal transition

## References

[1] Sung H, Ferlay J, Siegel RL (2021). Global Cancer statistics 2020: GLOBOCAN estimates of incidence and mortality worldwide for 36 cancers in 185 countries. CA Cancer J Clin.

[2] Hu Z, Li M, Chen Z (2019). Advances in clinical trials of targeted therapy and immunotherapy of lung cancer in 2018. Transl Lung Cancer Res.

[3] Nadal E, Massuti B, Domine M (2019). Immunotherapy with checkpoint inhibitors in non-small cell lung cancer: insights from long-term survivors. Cancer Immunol Immunother.

[4] Hirsch FR, Scagliotti GV, Mulshine JL (2017). Lung cancer: current therapies and new targeted treatments. Lancet.

[5] Gettinger S, Horn L, Jackman D (2018). Five-year Follow-up of Nivolumab in previously treated advanced non-small-cell lung Cancer: results from the CA209-003 study. J Clin Oncol.

[6] Uramoto H, Tanaka F (2014). Recurrence after surgery in patients with NSCLC. Transl Lung Cancer Res.

[7] Wu F, Fan J, He Y (2021). Single-cell profiling of tumor heterogeneity and the microenvironment in advanced non-small cell lung cancer. Nat Commun.

[8] Altorki NK, Markowitz GJ, Gao D (2019). The lung microenvironment: an important regulator of tumour growth and metastasis. Nat Rev Cancer.

[9] Lu T, Yang X, Shi Y (2020). Single-cell transcriptome atlas of lung adenocarcinoma featured with ground glass nodules. Cell Discov.

[10] Cassetta L, Pollard JW (2018). Targeting macrophages: therapeutic approaches in cancer. Nat Rev Drug Discov.

[11] Wagner J, Rapsomaniki MA, Chevrier S (2019). A single-cell atlas of the tumor and immune ecosystem of human breast Cancer. Cell.

[12] Chevrier S, Levine JH, Zanotelli V (2017). An immune atlas of clear cell renal cell carcinoma. Cell.

[13] Guo J, Yan Y, Yan Y (2017). Tumor-associated macrophages induce the expression of FOXQ1 to promote epithelial-mesenchymal transition and metastasis in gastric cancer cells. Oncol Rep.

[14] Hu Z, Jin X, Hong W (2023). Dissecting the single-cell transcriptome network of macrophage and identifies a signature to predict prognosis in lung adenocarcinoma. Cell Oncol (Dordr).

[15] Lewis CE, Pollard JW (2006). Distinct role of macrophages in different tumor microenvironments. Cancer Res.

[16] Wang Z, Zhao Y, Xu H (2020). CtBP1 promotes tumour-associated macrophage infiltration and progression in non-small-cell lung cancer. J Cell Mol Med.

[17] Guo Z, Song J, Hao J (2019). M2 macrophages promote NSCLC metastasis by upregulating CRYAB. Cell Death Dis.

[18] Zhang J, Cao J, Ma S (2014). Tumor hypoxia enhances non-small cell lung Cancer metastasis by selectively promoting macrophage M2 polarization through the activation of ERK signaling. Oncotarget.

[19] Zhang S, Che D, Yang F (2017). Tumor-associated macrophages promote tumor metastasis via the TGF-beta/SOX9 axis in non-small cell lung cancer. Oncotarget.

[20] Cardoso AP, Pinto ML, Pinto AT (2015). Matrix metalloproteases as maestros for the dual role of LPS- and IL-10-stimulated macrophages in cancer cell behaviour. BMC Cancer.

[21] Schafer ZT, Brugge JS (2007). IL-6 involvement in epithelial cancers. J Clin Invest.

[22] Shintani Y, Fujiwara A, Kimura T (2016). IL-6 secreted from Cancer-associated fibroblasts mediates Chemoresistance in NSCLC by increasing epithelial-mesenchymal transition signaling. J Thorac Oncol.

[23] Grivennikov SI, Greten FR, Karin M (2010). Immunity, inflammation, and cancer. Cell.

[24] Yan HQ, Huang XB, Ke SZ (2014). Interleukin 6 augments lung cancer chemotherapeutic resistance via ataxia-telangiectasia mutated/NF-kappaB pathway activation. Cancer Sci.

[25] Chang CH, Hsiao CF, Yeh YM (2013). Circulating interleukin-6 level is a prognostic marker for survival in advanced nonsmall cell lung cancer patients treated with chemotherapy. Int J Cancer.

[26] Yao X, Huang J, Zhong H (2014). Targeting interleukin-6 in inflammatory autoimmune diseases and cancers. Pharmacol Ther.

[27] Niu H, Huang Y, Yan L (2020). Knockdown of SMAD3 inhibits the growth and enhances the radiosensitivity of lung adenocarcinoma via p21 in vitro and in vivo. Int J Biol Sci.

[28] Johnson DE, O’Keefe RA, Grandis JR (2018). Targeting the IL-6/JAK/STAT3 signalling axis in cancer. Nat Rev Clin Oncol.

[29] Lee SM, Schulz C, Prabhash K (2023). First-line atezolizumab monotherapy versus single-agent chemotherapy in patients with non-small-cell lung cancer ineligible for treatment with a platinum-containing regimen (IPSOS): a phase 3, global, multicentre, open-label, randomised controlled study. Lancet.

[30] Restle D, Dux J, Li X (2023). Organ-specific heterogeneity in tumor-infiltrating immune cells and cancer antigen expression in primary and autologous metastatic lung adenocarcinoma. J Immunother Cancer.

[31] Kim N, Kim HK, Lee K (2020). Single-cell RNA sequencing demonstrates the molecular and cellular reprogramming of metastatic lung adenocarcinoma. Nat Commun.

[32] Fan Z, Jiang H, Wang Z (2016). Atorvastatin partially inhibits the epithelial-mesenchymal transition in A549 cells induced by TGF-beta1 by attenuating the upregulation of SphK1. Oncol Rep.

[33] Lamouille S, Xu J, Derynck R (2014). Molecular mechanisms of epithelial-mesenchymal transition. Nat Rev Mol Cell Biol.

[34] Ninsontia C, Phiboonchaiyanan PP, Chanvorachote P (2016). Zinc induces epithelial to mesenchymal transition in human lung cancer H460 cells via superoxide anion-dependent mechanism. Cancer Cell Int.

[35] Li S, Xu F, Zhang J (2018). Tumor-associated macrophages remodeling EMT and predicting survival in colorectal carcinoma. Oncoimmunology.

[36] Zugowski C, Lieder F, Muller A (2011). STAT3 controls matrix metalloproteinase-1 expression in colon carcinoma cells by both direct and AP-1-mediated interaction with the MMP-1 promoter. Biol Chem.

[37] Sullivan NJ, Sasser AK, Axel AE (2009). Interleukin-6 induces an epithelial-mesenchymal transition phenotype in human breast cancer cells. Oncogene.

[38] An J, Gu Q, Cao L (2021). Serum IL-6 as a vital predictor of severe lung cancer. Ann Palliat Med.

[39] Philips RL, Wang Y, Cheon H (2022). The JAK-STAT pathway at 30: much learned, much more to do. Cell.

[40] Zhou J, Qu Z, Yan S (2015). Differential roles of STAT3 in the initiation and growth of lung cancer. Oncogene.

[41] Jing B, Wang T, Sun B (2020). IL6/STAT3 signaling orchestrates Premetastatic niche formation and immunosuppressive traits in lung. Cancer Res.

[42] Au-Yeung N, Horvath CM (2018). Transcriptional and chromatin regulation in interferon and innate antiviral gene expression. Cytokine Growth Factor Rev.

[43] Platanitis E, Decker T (2018). Regulatory networks involving STATs, IRFs, and NFkappaB in inflammation. Front Immunol.

[44] Ramji DP, Foka P (2002). CCAAT/enhancer-binding proteins: structure, function and regulation. Biochem J.

[45] Lu T, Li M, Zhao M (2022). Metformin inhibits human non-small cell lung cancer by regulating AMPK-CEBPB-PDL1 signaling pathway. Cancer Immunol Immunother.

