# Supplementary material for: IL6-STAT3-C/EBPβ-IL6 positive feedback loop in tumor-associated macrophages promotes the EMT and metastasis of lung adenocarcinoma
Source: J Exp Clin Cancer Res. 2024 Feb 29;43:63. doi: 10.1186/s13046-024-02989-x (PMC10903044; doi:10.1186/s13046-024-02989-x)
Supplement: Supplementary file 1 — Supplementary material 1. [file 13046_2024_2989_MOESM1_ESM.zip › Figure legend.docx]

Figure 1 Annotation and function of myeloid cells and macrophages by scRNA-seq in tumor and normal tissue. a) UMAP plot colored by different clusters. b) Dot plot of mean expression of myeloid cells and macrophages' characteristic genes in different clusters. c) UMAP plot colored by different cell types. d) Complex heatmap of selected marker genes in each cell cluster. Up: Tissue preference of each cluster. Down: Relative expression of marker genes associated with each cell subset. e) Relative contribution of each cell type in normal vs. tumor tissue and early vs. advanced LUAD. f) Kaplan–Meier curves of survival analysis in TCGA LUAD patients based on the TAMs infiltration. g) Left: Difference genes between normal tissue, early LUAD, and advanced LUAD, with a threshold of |log2FC| > 0.5 & p_val_adj < 0.05, and take the intersection of the differential genes of the three groups. Right: Heatmap of 26 genes that differ among the three groups. h) Immunofluorescence shows the expression of TAMs marker and IL-6 (Red CD68, Pink CD163, Green IL-6, Blue Dapi), scale bar represents 50μm.

Figure 2 TAMs promote LUAD cell proliferation, migration and invasion by secreting IL-6. a) Cell migration and invasion ability of LUAD cells (A549 and H358) alone, stimulated with IL-6 or stimulated with IL-6 followed by addition of IL-6 neutralizing antibody was determined by the transwell assay. b) Cell proliferation ability of LUAD cells (A549 and H358) alone, stimulated with IL-6 or stimulated with IL-6 followed by addition of IL-6 neutralizing antibody was determined by the CCK8 assay. c) The tumor size and tumor weight in the A549 alone, A549 + IL-6 stimulation and A549 + IL-6 stimulation + IL-6-NA groups. d) RT-qPCR analyzed the expression of the most often released cytokines by macrophages after IL-6 stimulation. e) Elisa analysis showed changes in macrophage IL-6 expression over time after IL-6 stimulation. f & g) Cell migration, invasion and proliferation ability of LUAD cells (A549 and H358) alone, co-culture with M2-like macrophages or co-culture with M2-like macrophages and stimulated with IL-6 was determined by the transwell assay and CCK8 assay.

Figure 3 IL-6 promotes LUAD progression by activating the JAK2/STAT3 pathway in M2-like macrophages. a) GSVA analysis of differential pathways between AMs, TAMs in early and advanced LUAD. b) Effects of IL-6 stimulation on common pathway expression in TAMs detected by western blot analysis. c) Expression of the JAK2/STAT3 pathway with IL-6-stimulated, IL-6-stimulated with the addition of IL-6-NA in M2-like macrophages were analyzed by western blot. d) Effect of AZD1480 and WP1066 on JAK2/STAT3 pathway expression in THP1 derived M2-like macrophages after IL-6 stimulation were analyzed by western blot. e) Cell migration and invasion ability of LUAD cells alone, co-culture with M2-like macrophages or co-culture with M2-like macrophages followed by addition of AZD1480 and WP1066 was determined by the transwell assay. f) RT-qPCR and Elisa analysis showed IL-6 expression in M2-like macrophages after IL-6 stimulation or IL-6 stimulation with the addition of AZD1480 and WP1066 (Left: Elisa results. Right: RT-qPCR results). g) IL-6 expression after STAT3 overexpression or knockdown was analyzed by western blot. h) RT-qPCR and Elisa analysis showed IL-6 expression in M2-like macrophages after STAT3 overexpression or knockdown.

Figure 4 pSTAT3 promotes tumor progression by regulating C/EBPβ expression in TAMs. a) Cell migration and invasion ability of LUAD cells, C/EBPβ overexpression or C/EBPβ knockdown was determined by the transwell assay. b) Effects of IL-6 stimulation on C/EBPβ expression in TAMs. c) Expression of C/EBPβ after STAT3 overexpression or knockdown. d) RT-qPCR and Elisa analysis showed IL-6 expression in M2-like macrophages after C/EBPβ overexpression or knockdown. e) Expression of pSTAT3 and C/EBPβ in the nucleus after IL-6 stimulation. f) The localization of pSTAT3 and C/EBPβ were analyzed by immunofluorescence staining. The nuclei were stained with 4',6-diamidino-2-phenylindole (DAPI; blue). The line charts represent fluorescence intensity (MFI), presenting the distance from α to β, scale bar represents 100μm.

Figure 5 Regulatory role of STAT3/ C/EBPβ/IL-6. a) C/EBPβ and IL-6 expression after STAT3 knockdown or overexpression. b) STAT3 and IL-6 expression after C/EBPβ knockdown or overexpression. c) IL-6 expression after STAT3 knockdown and C/EBPβ overexpression or STAT3 overexpression and C/EBPβ knockdown. d) IL-6 expression after STAT3 knockdown and rescued by IL-6 stimulation. e) IL-6 expression after C/EBPβ knockdown and rescued by IL-6 stimulation.

Figure 6 Mechanisms of STAT3 regulation of C/EBPβ and C/EBPβ regulation of IL-6. a) ChIP assay demonstrated the binding of STAT3 to the C/EBPβ promoter in THP1-derived M2-like macrophages. b) RT-qPCR of the ChIP products confirmed the binding capacity of STAT3 to the C/EBPβ promoter. c) Selective mutation analyses identified STAT3-responsive regions in the C/EBPβ promoter. d) Luciferase reporter plasmids with wild and mutated C/EBPβ promoters were transfected into 293T cells. Relative luciferase activity was determined using IL-6 stimulation or STAT3 overexpression. e) ChIP assay demonstrated the binding of C/EBPβ to the IL-6 promoter in. f) RT-qPCR of the ChIP products confirmed the binding capacity of C/EBPβ to the IL-6 promoter. g) Selective mutation analyses identified C/EBPβ-responsive regions in the IL-6 promoter. h) Luciferase reporter plasmids with wild and mutated IL-6 promoters were transfected into 293T cells. Relative luciferase activity was determined using IL-6 stimulation, C/EBPβ or STAT3 overexpression.

Figure 7 IL-6 promotes LUAD migration and invasion through activation of EMT pathway in vivo and in vitro. a) Expression of EMT-related genes after co-culture with M2-like macrophages. b) Expression of EMT-related genes using IL-6 stimulation or IL-6-neutralising antibodies. c) IHC analyzed the expression of CD206, IL-6, E-cadherin, N-cadherin, Vimentin and Snail, and their relationships of tumors from LUAD patients. d) The tumor size and tumor weight in the A549 alone, A549 + control TAMs, A549 + IL-6 overexpression TAMs, A549 + IL-6 knockout TAMs, and A549 + TAMs + IL-6 neutralizing antibodies groups. e) IHC analyzed the expression of E-cadherin, N-cadherin, Vimentin and Snail protein of tumors from the subgroups above.

Supplementary Figure 1 a) UMAP plot colored by cells of different origins. b) UMAP plot colored by cells of different dataset. c) Relative contribution of each cell type in normal vs. tumor tissue, and in early vs. advanced LUAD. d) UMAP plot colored by cell types and FeaturePlot of different cell types expressing IL-6. e) Correlation between TAMs-specific markers CD68, CD163 with IL-6.

Supplementary Figure 2 a) Kaplan–Meier curves of survival analysis in TCGA LUAD patients based on IL-6 expression. b) The morphological characteristics of tumor xenograft in the A549 alone, A549 + IL-6 stimulation and A549 + IL-6 stimulation + IL-6-NA groups. c) IL-6R and GP130 expression in TAMs and macrophages using the scRNA-seq data. d) RT-qPCR analysis of macrophages IL-6 mRNA over time after IL-6 stimulation (Left: THP1 derived M2-like macrophage. Right: RAW264.7 derived M2-like macrophage).

Supplementary Figure 3 a) Schema for representing the experiment procedures of co-culture of macrophages with tumor cells. b) Expression of the M2-like macrophage marker CD206 after THP1 polarization detected by flow cytometry (Left: flow cytometry gate strategy, Right: proportion of CD11b+CD206+ cells). c) Expression of the M2-like macrophage marker CD206 after BMDM polarization detected by flow cytometry (Left: flow cytometry gate strategy, Right: proportion of CD11b+CD206+ cells). d) RT-qPCR analyzed the expression of the markers of pan-macrophage, M1-like and M2-like macrophages.

Supplementary Figure 4 a) Effects of different concentrations of JAK2/STAT3 pathway inhibitor (AZD1480 and WP1066) on JAK2/STAT3 pathway expression in TAMs. b) Effect of AZD1480 and WP1066 on JAK2/STAT3 pathway expression in RAW264.7 derived M2-like macrophages after IL-6 stimulation were analyzed by Western blot. c) Cell migration and invasion ability of LUAD cells alone, co-culture with M2-like macrophages or co-culture with M2-like macrophages followed by addition of AZD1480 and WP1066 was determined by the transwell assay. d) RT-qPCR and Elisa analysis showed IL-6 expression in RAW264.7 derived M2-like macrophages after IL-6 stimulation or IL-6 stimulation with the addition of AZD1480 and WP1066 (Left: Elisa results. Right: RT-qPCR results). e) Cell migration, invasion abilities of A549 cells alone, or added the AZD1480 or WP1066 were determined by the transwell assay, scale bar represents 200μm.

Supplementary Figure 5 a) STAT3 mRNA expression after STAT3 overexpression plasmid or si-RNA transfection. b) ChIP assays were used to test whether STAT3 can bind directly to the IL-6 promoter in M2-like macrophages.

Supplementary Figure 6 a & b) Kaplan–Meier curves of overall survival and disease-free survival in TCGA LUAD patients based on C/EBPβ expression. c) C/EBPβ expression after C/EBPβ overexpression plasmid or si-RNA transfection was analyzed by western blot. d) Effects of IL-6 stimulation on C/EBPβ expression in TAMs. e) RT-qPCR showed C/EBPβ expression in M2-like macrophages after IL-6 stimulation. f) Correlation of IL-6 and C/EBPβ expression in TCGA database.

Supplementary Figure 7 a) RT-qPCR analysis of LUAD cell EMT and MMPs mRNA over time after IL-6 stimulation. b) Expression of EMT-related genes of BEAS-2B or A549 cells after co-culture with M2-like macrophages, and cell migration, invasion abilities of A549 cells or BEAS-2B cells alone, co-culture with M2-like macrophages were determined by the transwell assay, scale bar represents 200μm. c) Correlation between M2-like macrophage markers and EMT-related genes in LUAD patient. d) The morphological characteristics of tumor xenograft in the A549 alone, A549 + control TAMs, A549 + IL-6 overexpression TAMs, A549 + IL-6 knockout TAMs, and A549 + TAMs + IL-6 neutralizing antibodies groups.
