# Supplementary material for: IL6-STAT3-C/EBPβ-IL6 positive feedback loop in tumor-associated macrophages promotes the EMT and metastasis of lung adenocarcinoma
Source: J Exp Clin Cancer Res. 2024 Feb 29;43:63. doi: 10.1186/s13046-024-02989-x (PMC10903044; doi:10.1186/s13046-024-02989-x)
Supplement: Supplementary file 1 — Supplementary material 1. [file 13046_2024_2989_MOESM1_ESM.zip › primer.docx]

CCL22 F：ATCGCCTACAGACTGCACTC

R：GACGGTAACGGACGTAATCAC

CD206 F：GGGTTGCTATCACTCTCTATGC

R：TTTCTTGTCTGTTGCCGTAGTT

CCL18 F：TCTATACCTCCTGGCAGATTC

R：TTTCTGGACCCACTTCTTATTG

IL6 F: CCAGGAGCCCAGCTATGAAC

R: CCCAGGGAGAAGGCAACTG

STAT3 F: ATGGCCCAATGGAATCAGC

R: TCACATGGGGGAGGTAGCGC

CEBPB F: GCACAGCGACGAGTACAAGA

R: TGCTTGAACAAGTTCCGCAG

ACTB F: TGACGTGGACATCCGCAAAG

R: CTGGAAGGTGGACAGCGAGG

Actin F：GGTGGGAATGGGTCAGAAGG

R：GTTGGCCTTAGGGTTCAGGG

Il6 F：TAGTCCTTCCTACCCCAATTTCC

R：TTGGTCCTTAGCCACTCCTTC

Cebpb F：CAAGCTGAGCGACGAGTACA

R：AGCTGCTCCACCTTCTTCTG

Stat3 F：AATGGAAATTGCCCGGATCG

R：TCCTGAAGATGCTGCTCCAA

Arg1 F：CTCCAAGCCAAAGTCCTTAGAG

R：AGGAGCTGTCATTAGGGACATC

Cd206 F：GAGGGAAGCGAGAGATTATGGA

R：GCCTGATGCCAGGTTAAAGCA

Cd163 F：ATGGGTGGACACAGAATGGTT

R：CAGGAGCGTTAGTGACAGCAG

Il10 F：CTTACTGACTGGCATGAGGATCA

R：GCAGCTCTAGGAGCATGTGG

CSF2 F: TCCTGAACCTGAGTAGAGACAC

R: TGCTGCTTGTAGTGGCTGG

TNF F: GAGGCCAAGCCCTGGTATG

R: CGGGCCGATTGATCTCAGC

CCL22 F: ATTACGTCCGTTACCGTCTGC

R: TCCCTGAAGGTTAGCAACACC

CD40 F: TTGGGGTCAAGCAGATTGCTA

R: GCAGATGACACATTGGAGAAGA

IL10 F: GACTTTAAGGGTTACCTGGGTTG

R: TCACATGCGCCTTGATGTCTG

IL1B F: ATGATGGCTTATTACAGTGGCAA

R: GTCGGAGATTCGTAGCTGGA

CCL2 F: CAGCCAGATGCAATCAATGCC

R: TGGAATCCTGAACCCACTTCT

CXCL2 F: ATCCAAAGTGTGAAGGTGAAGTC

R: CCCATTCTTGAGTGTGGCTATG

IFNG F: TCGGTAACTGACTTGAATGTCCA

R: TCGCTTCCCTGTTTTAGCTGC

IL4 F: CCAACTGCTTCCCCCTCTG

R: TCTGTTACGGTCAACTCGGTG

CXCL8 F: ACTGAGAGTGATTGAGAGTGGAC

R: AACCCTCTGCACCCAGTTTTC

IFNA F: GCCTCGCCCTTTGCTTTACT

R: CTGTGGGTCTCAGGGAGATCA

IFNB F: ATGACCAACAAGTGTCTCCTCC

R: GGAATCCAAGCAAGTTGTAGCTC

Vimentin F: AGTCCACTGAGTACCGGAGAC

R: CATTTCACGCATCTGGCGTTC

N-Cadherin F: TGCGGTACAGTGTAACTGGG

R: GAAACCGGGCTATCTGCTCG

β-Catenin F: CATCTACACAGTTTGATGCTGCT

R: GCAGTTTTGTCAGTTCAGGGA

Snail F: TCGGAAGCCTAACTACAGCGA

R: AGATGAGCATTGGCAGCGAG

E-Cadherin F: ATTTTTCCCTCGACACCCGAT

R: TCCCAGGCGTAGACCAAGA

MMP2 F: TACAGGATCATTGGCTACACACC

R: GGTCACATCGCTCCAGACT

MMP7 F: GAGTGAGCTACAGTGGGAACA

R: CTATGACGCGGGAGTTTAACAT

MMP9 F: TGTACCGCTATGGTTACACTCG

R: GGCAGGGACAGTTGCTTCT

STAT3 to CEBPB ChIP primer

P1 F: CAGCAAGACTGGCAGGCAAGAT

R: AAGGCAGCTCCTCAGGTCTCAG (-1963 - -1799bp)

P2 F: TAATGGTGGCTGGCGATA

R: AGAGCTGGAGAAATGACATC (-1645 - -1358bp)

P3 F: TAGGAGTGGCAGAGAGGT

R: GCCTGGACTTGAACACAG (-1217 - -1072bp)

P4 F: GTTGTCGCTTTCCCGTCTGTGA

R: CAAGTCCCTGCCCTGTGCTTAC (-1067 - -956bp)

P5 F: CACAGGGCAGGGACTTGGTAAA

R: CTGAAGTTGCTAGGGAGGGACT (-973 - -867bp)

P6 F: GTCGTCACAGGCGTCAAGTCTT

R: GGTGGCCGCTATTAGTGAGG ( -337 +250bp)

STAT3 to IL6 ChIP primer

P1 F: GGTCCTTGATGTAACAGCCAGG

R: AAGTGTCTCCTTCCTCTCCTGG (-2000 – 1856bp)

P2 F: GCCAGGAGAGGAAGGAGACACT

R: GCGGTGGGATTCTTTGGTGTGA (-1880 - 1497bp)

P3 F: GGCGGGTCCTGAAATGTTATGC

R: GGTTGTCCCTCCAGTCTCCAGA (-1433 - 1218bp)

P4 F: GCTGCGATGGAGTCAGAGGAA

R: AGCTGAAGTCATGCACGAAGTT (-496 - -322bp)

P5 F: GCCTCAATGACGACCTAAGCTG

R: AGCCTCAGACATCTCCAGTCCT (-223 - -2bp)

CEBPB to IL6 ChIP primer

P1 F GCCAGGAGAGGAAGGAGACACT

R CCTGCATGAAACGAAGCCACTG (-1879 - -1618bp)

P2 F GCCAGGAGAGGAAGGAGACACT

R GCGGTGGGATTCTTTGGTGTGA (-1879 - -1497bp)

P3 F CTGGGAGCAGTGGCTTCGTTTC

R GCGGTGGGATTCTTTGGTGTGA (-1639 - -1504bp)

P4 F GGCGGGTCCTGAAATGTTATGC

R GGTTGTCCCTCCAGTCTCCAGA (-1432 - -1213bp)

P5 F GCAGCAGCCAACCTCCTCTAA

R GCAGTTACTTCAAGGCGTCTCC (-723 - -617bp)

P6 F GCCTCAATGACGACCTAAGCTG

R AGCCTCAGACATCTCCAGTCCT (-222 - -1bp)
