# Supplementary figures and images for: IL6-STAT3-C/EBPβ-IL6 positive feedback loop in tumor-associated macrophages promotes the EMT and metastasis of lung adenocarcinoma
Source: J Exp Clin Cancer Res. 2024 Feb 29;43:63. doi: 10.1186/s13046-024-02989-x (PMC10903044; doi:10.1186/s13046-024-02989-x)

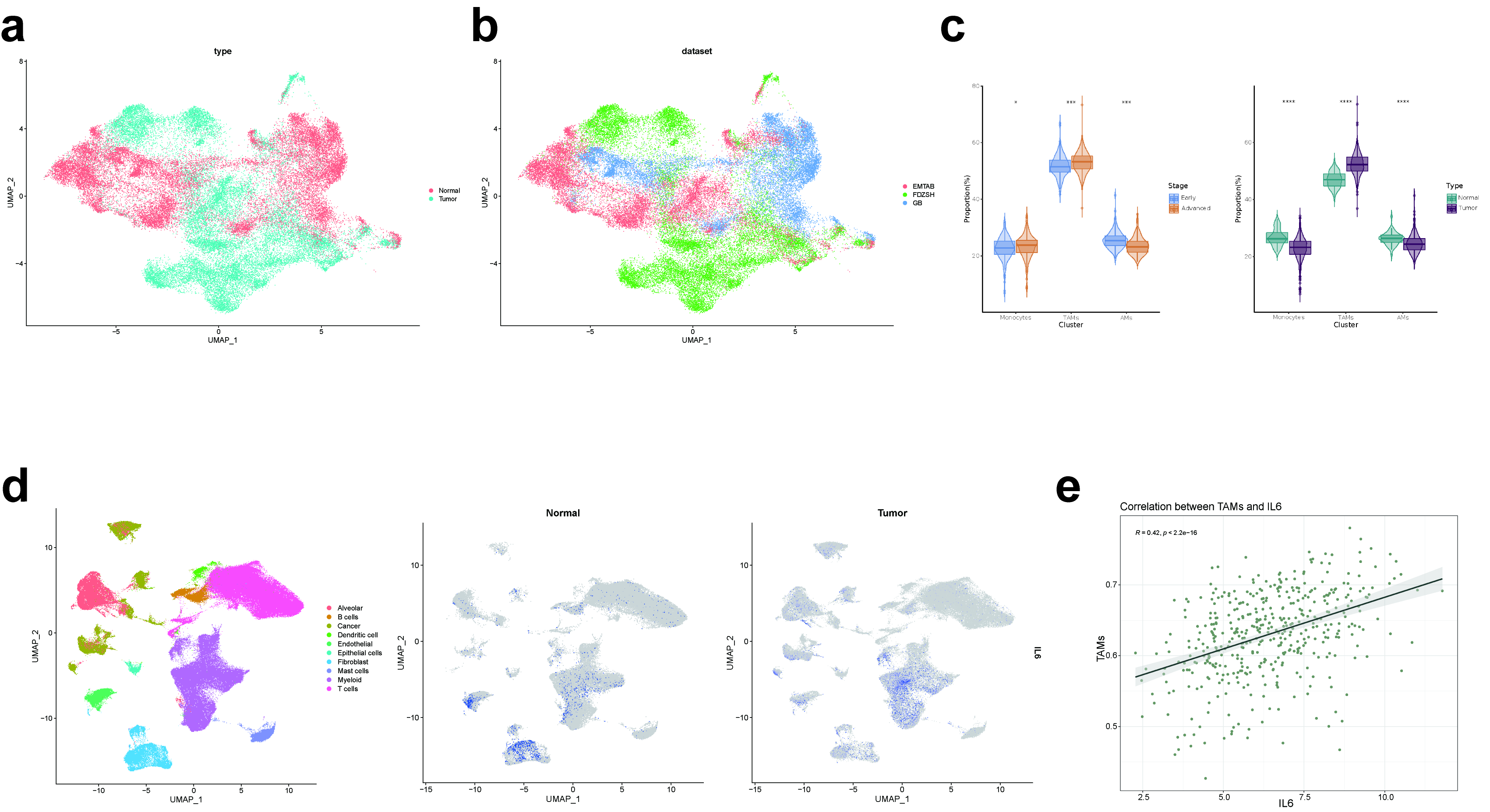

Supplement: Supplementary file 1 — Supplementary material 1. [file 13046_2024_2989_MOESM1_ESM.zip › Fig. S1.tif]

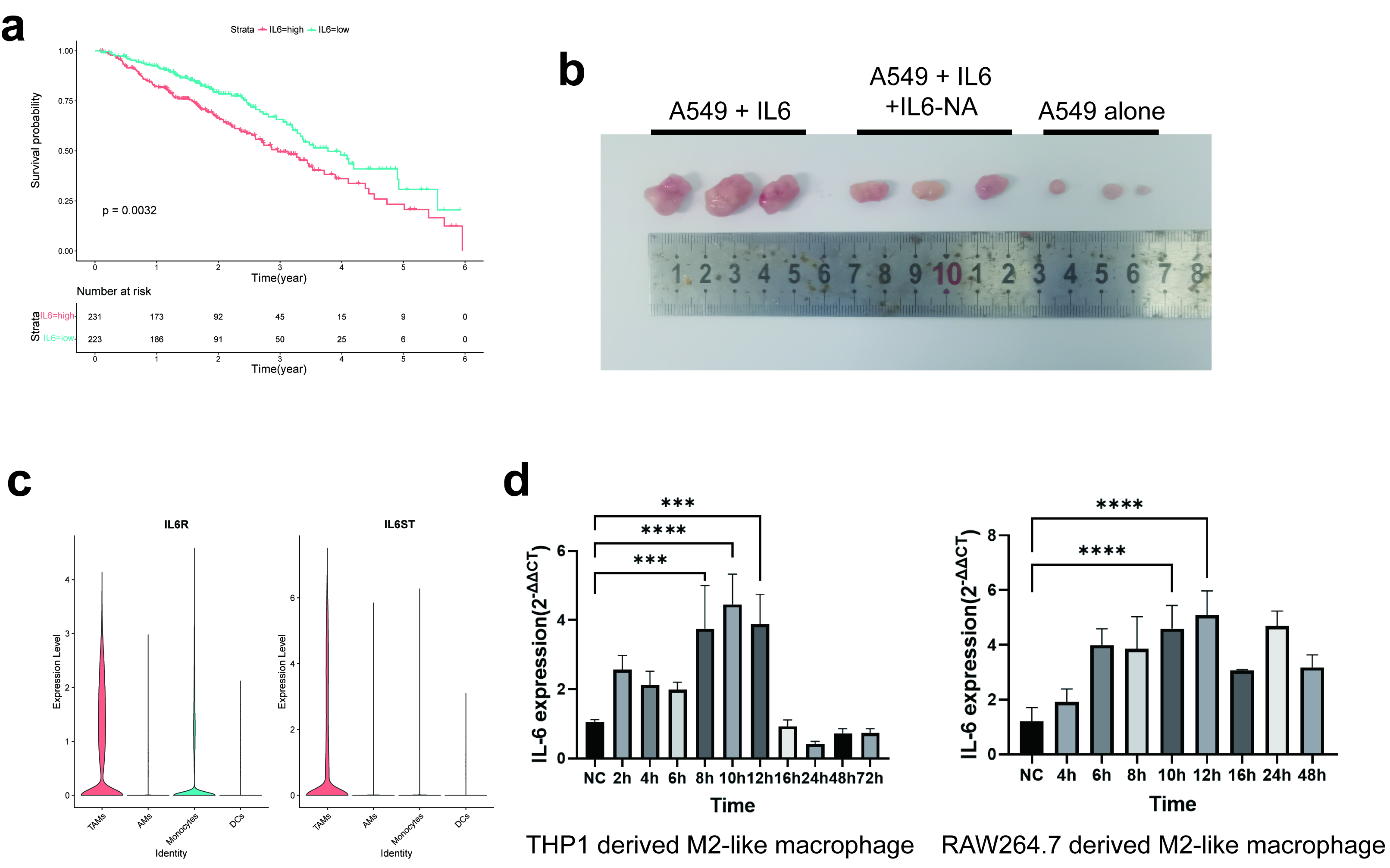

Supplement: Supplementary file 1 — Supplementary material 1. [file 13046_2024_2989_MOESM1_ESM.zip › Fig. S2.tif]

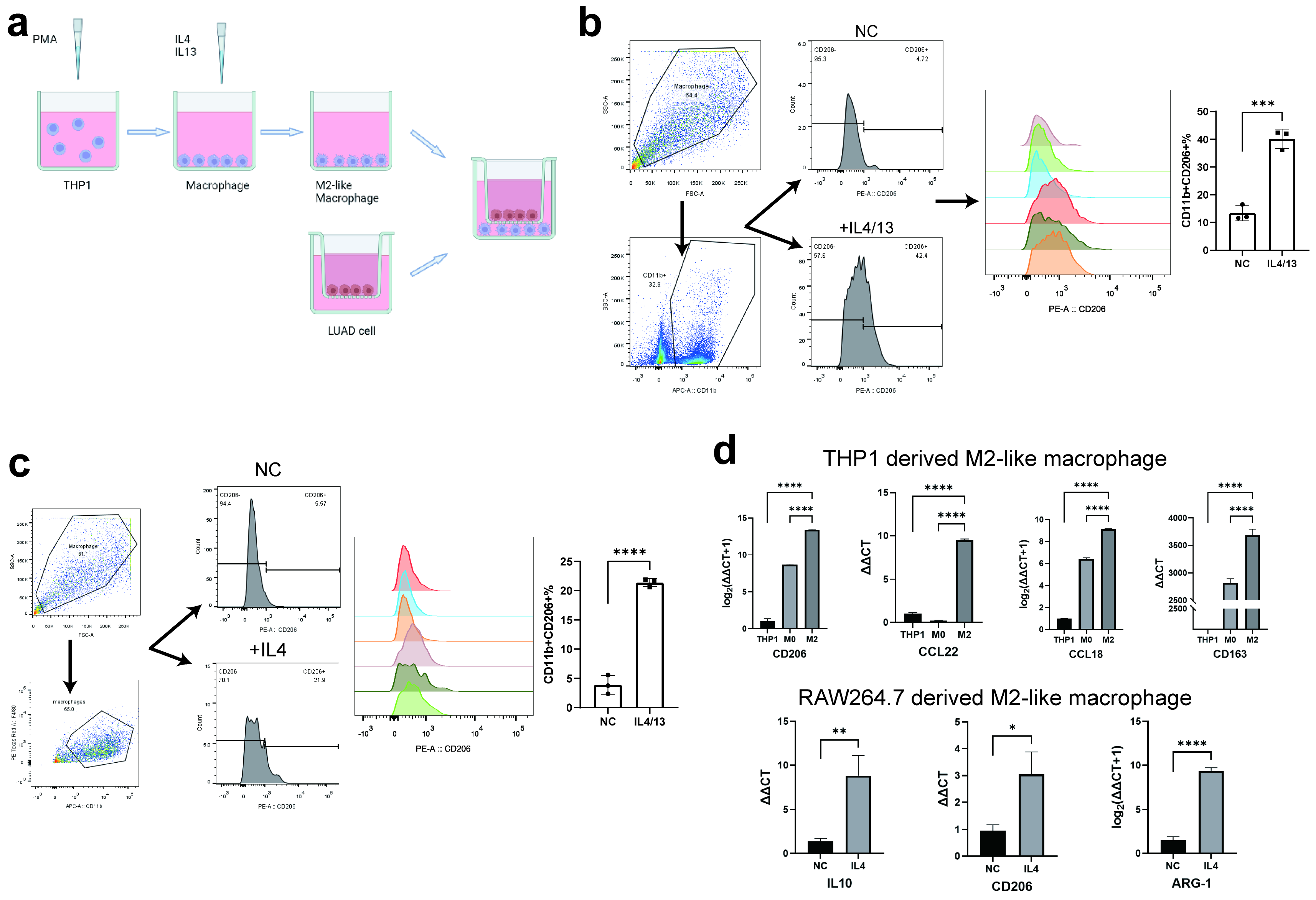

Supplement: Supplementary file 1 — Supplementary material 1. [file 13046_2024_2989_MOESM1_ESM.zip › Fig. S3.tif]

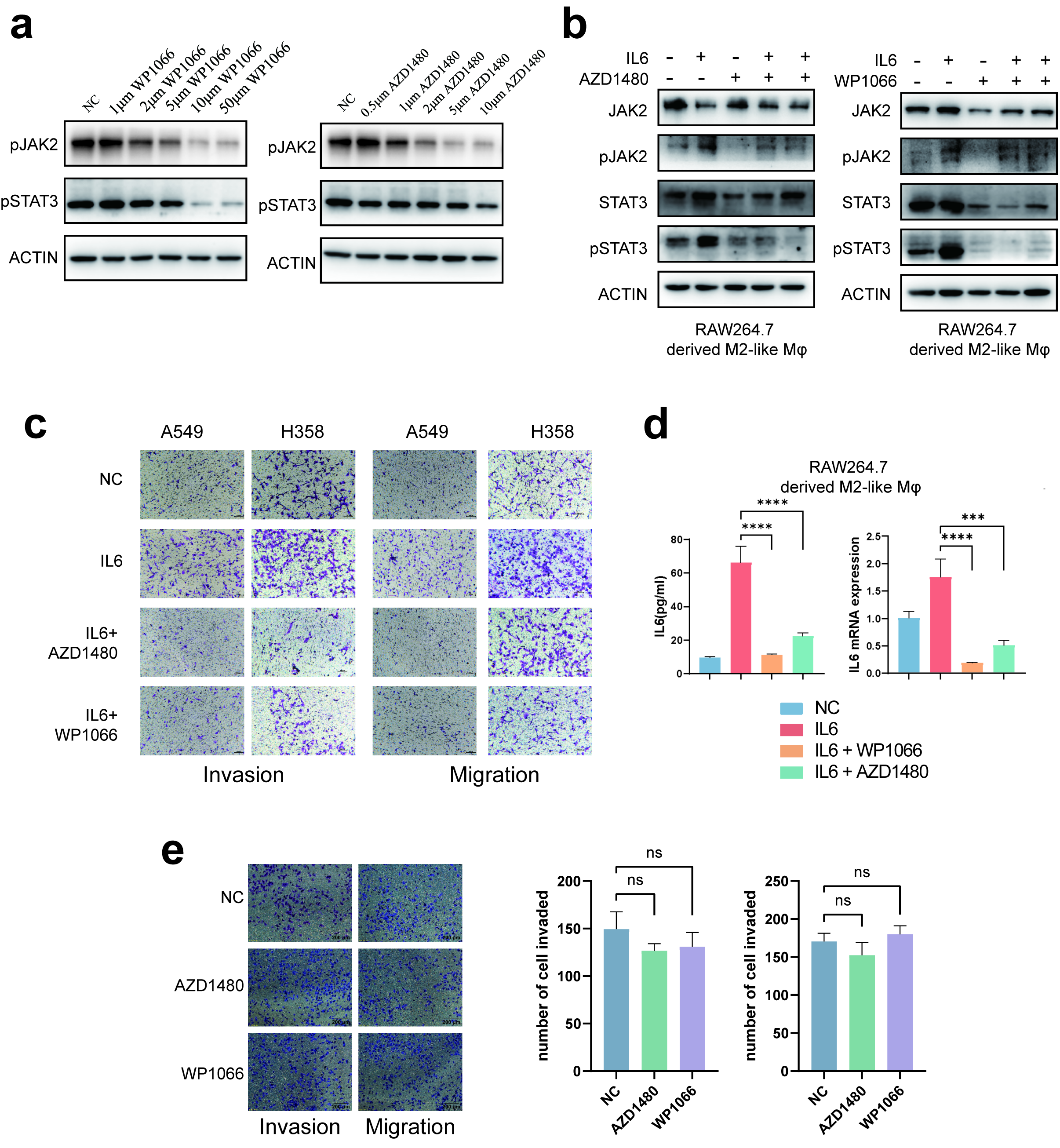

Supplement: Supplementary file 1 — Supplementary material 1. [file 13046_2024_2989_MOESM1_ESM.zip › Fig. S4.tif]

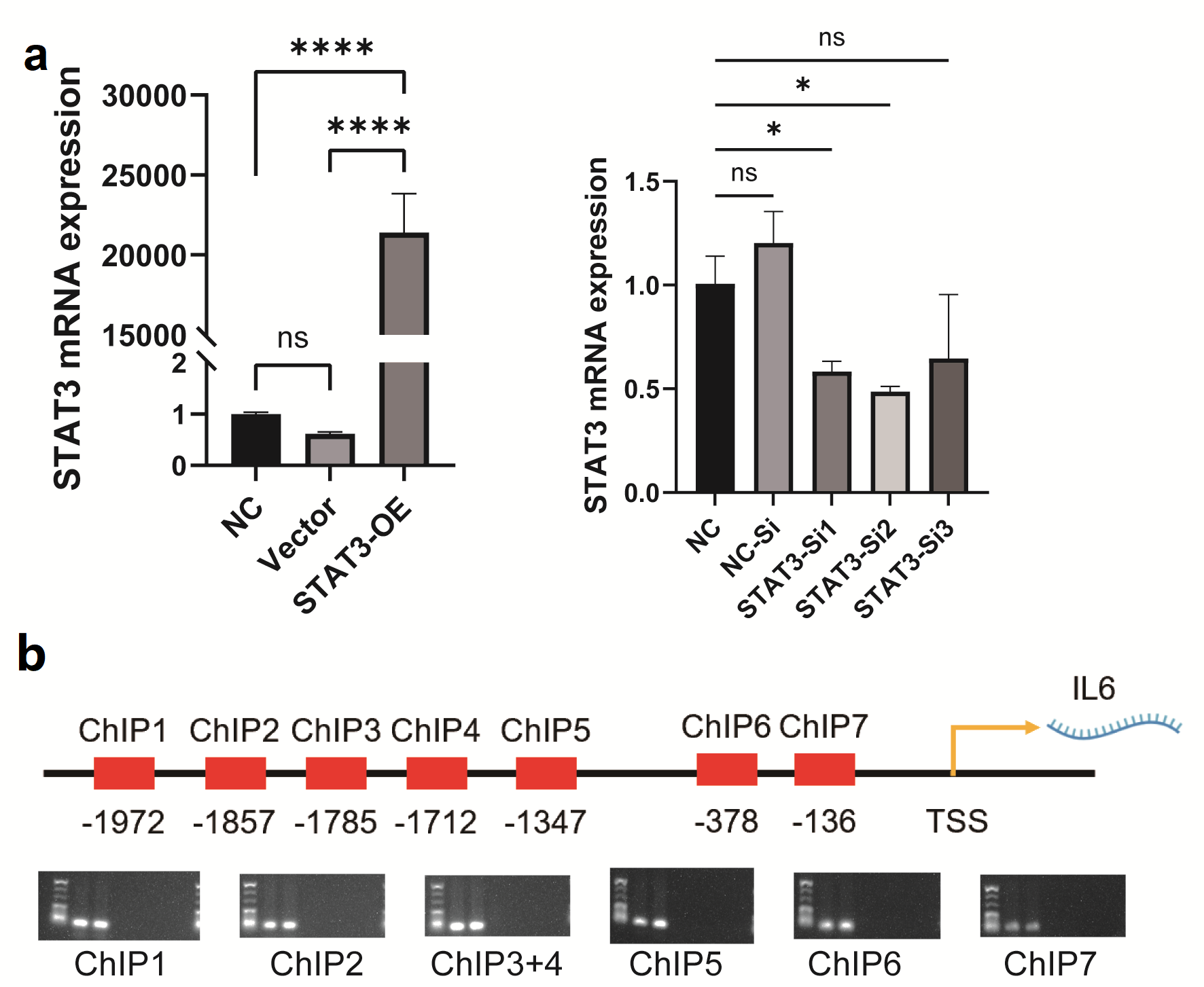

Supplement: Supplementary file 1 — Supplementary material 1. [file 13046_2024_2989_MOESM1_ESM.zip › Fig. S5.tif]

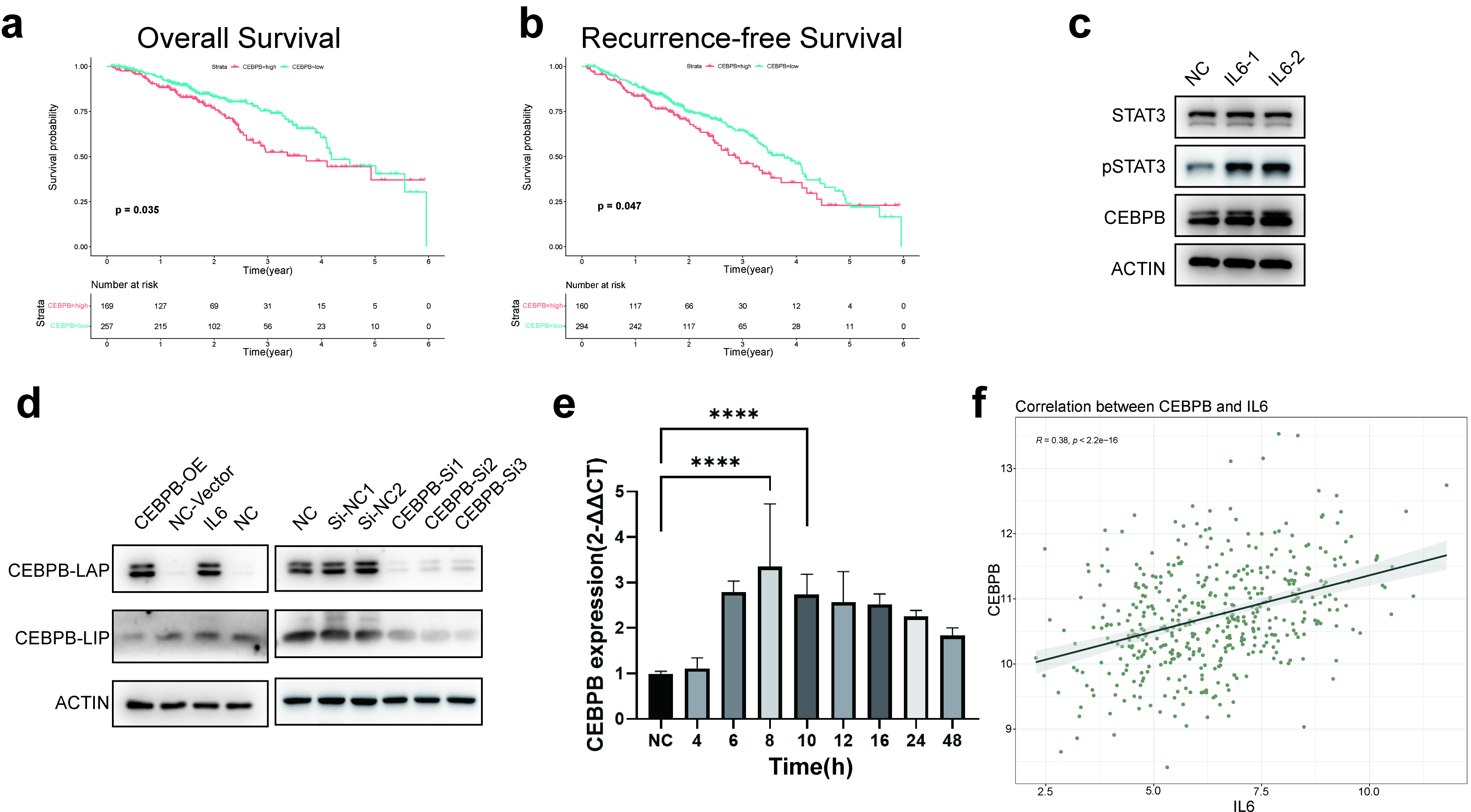

Supplement: Supplementary file 1 — Supplementary material 1. [file 13046_2024_2989_MOESM1_ESM.zip › Fig. S6.tif]

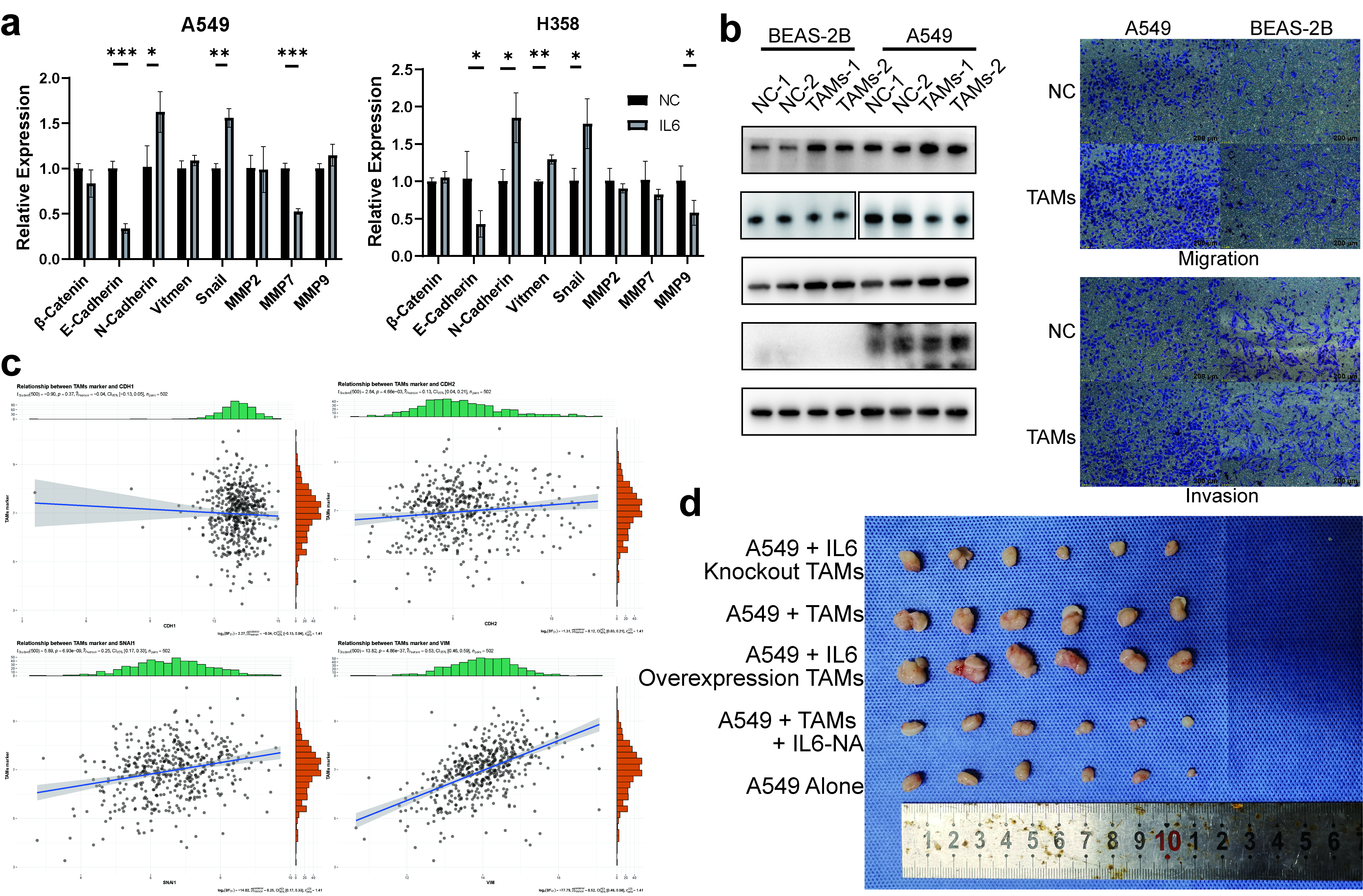

Supplement: Supplementary file 1 — Supplementary material 1. [file 13046_2024_2989_MOESM1_ESM.zip › Fig. S7.tif]
